# Harnessing single-cell genomics to improve the physiological fidelity of organoid-derived cell types

**DOI:** 10.1186/s12915-018-0527-2

**Published:** 2018-06-05

**Authors:** Benjamin E. Mead, Jose Ordovas-Montanes, Alexandra P. Braun, Lauren E. Levy, Prerna Bhargava, Matthew J. Szucs, Dustin A. Ammendolia, Melanie A. MacMullan, Xiaolei Yin, Travis K. Hughes, Marc H. Wadsworth, Rushdy Ahmad, Seth Rakoff-Nahoum, Steven A. Carr, Robert Langer, James J. Collins, Alex K. Shalek, Jeffrey M. Karp

**Affiliations:** 1 Division of Engineering in Medicine, Department of Medicine, Brigham & Women’s Hospital, Harvard Medical School, Boston, MA USA; 2 Harvard-MIT Division of Health Sciences & Technology, Cambridge, MA USA; 3 Koch Institute for Integrative Cancer Research, MIT, Cambridge, MA USA; 4 Harvard Stem Cell Institute, Cambridge, MA USA; 5 Broad Institute of Harvard and MIT, Cambridge, MA USA; 6 Institute for Medical Engineering and Science, MIT, Cambridge, MA USA; 7 Department of Chemistry, MIT, Cambridge, MA USA; 8 Department of Chemical Engineering, MIT, Cambridge, MA, USA; 9 Ragon Institute of MGH, MIT and Harvard, Cambridge, MA USA; 10 Divisions of Infectious Diseases and Gastroenterology, Boston Children’s Hospital and Harvard Medical School, Boston, MA USA; 11 Wyss Institute for Biologically Inspired Engineering, Harvard University, Boston, MA USA; 12 Department of Biological Engineering, MIT, Cambridge, MA USA; 13 Synthetic Biology Center, MIT, Cambridge, MA USA; 14 Center for Microbiome Informatics and Therapeutics, MIT, Cambridge, MA USA

**Keywords:** Single-cell RNA-seq, Chemical biology, Stem cell-derived models, Paneth cell, Intestinal organoid, Intestinal stem cell, Differentiation, Systems biology

## Abstract

**Background:**

Single-cell genomic methods now provide unprecedented resolution for characterizing the component cell types and states of tissues such as the epithelial subsets of the gastrointestinal tract. Nevertheless, functional studies of these subsets at scale require faithful in vitro models of identified in vivo biology. While intestinal organoids have been invaluable in providing mechanistic insights in vitro, the extent to which organoid-derived cell types recapitulate their in vivo counterparts remains formally untested, with no systematic approach for improving model fidelity.

**Results:**

Here, we present a generally applicable framework that utilizes massively parallel single-cell RNA-seq to compare cell types and states found in vivo to those of in vitro models such as organoids. Furthermore, we leverage identified discrepancies to improve model fidelity. Using the Paneth cell (PC), which supports the stem cell niche and produces the largest diversity of antimicrobials in the small intestine, as an exemplar, we uncover fundamental gene expression differences in lineage-defining genes between in vivo PCs and those of the current in vitro organoid model. With this information, we nominate a molecular intervention to rationally improve the physiological fidelity of our in vitro PCs. We then perform transcriptomic, cytometric, morphologic and proteomic characterization, and demonstrate functional (antimicrobial activity, niche support) improvements in PC physiology.

**Conclusions:**

Our systematic approach provides a simple workflow for identifying the limitations of in vitro models and enhancing their physiological fidelity. Using adult stem cell-derived PCs within intestinal organoids as a model system, we successfully benchmark organoid representation, relative to that in vivo, of a specialized cell type and use this comparison to generate a functionally improved in vitro PC population. We predict that the generation of rationally improved cellular models will facilitate mechanistic exploration of specific disease-associated genes in their respective cell types.

**Electronic supplementary material:**

The online version of this article (10.1186/s12915-018-0527-2) contains supplementary material, which is available to authorized users.

## Background

Intestinal organoids, derived from intestinal stem cells (ISCs) and composed of ISCs, Paneth cells (PCs), enteroendocrine cells (EECs), goblet cells, and absorptive enterocytes, have been invaluable to the study of intestinal biology [1]. Recent advances in massively parallel single-cell RNA-sequencing (scRNA-seq) [2] have enabled the cataloging of cell types and states of the murine small intestinal epithelium [3] and intestinal organoids [4], offering extensive insight into tissue heterogeneity, specifically within subsets of rare secretory cell populations. However, there have been no formal comparisons of how the in vitro intestinal organoid condition recapitulates the defined in vivo cell types. While the generation of comprehensive cellular atlases has become a major focus of a global effort to map tissues in humans, model organisms, and derived organoids at single-cell resolution [5], the challenge of how to functionally investigate key insights from cell types in vivo, or even more simply to confirm the high-fidelity representation of these states in existing model systems remains [6, 7].

Intestinal organoids are a compelling system with which to study specialized cells of the epithelium. They are self-organizing, stem cell-derived structures, which, to a reasonable degree, resemble their in vivo counterpart, can be rapidly grown, and are amenable to many biochemical and genetic perturbations [8]. Recent work has demonstrated the utility of organoids in assessing bulk phenotypes that are readily observed and easily selected for, such as phenotypes of cystic fibrosis and the study of cancer-associated mutational signatures [9, 10]. However, the application of organoid models to the study of complex disease, such as polygenic inflammatory disease, has been limited. In such instances, the subtler phenotypes, such as those present in inflammatory bowel disease (IBD), may not manifest if the originating cell state present in vivo is not accurately represented within an organoid model. This challenge is particularly clear in IBD [11], where loci identified through genome wide association studies (GWAS) have proven difficult to efficiently examine through the use of in vivo animal models.

For instance, PC dysfunction is implicated in Crohn’s disease, a subset of IBD typically afflicting the small bowel [12]. Co-localized with LGR5^+^ ISCs of the small intestinal crypts, long-lived PCs [13] support maintenance of the ISC niche, producing the Wnt and Notch signaling ligands WNT3 and DLL4 [14], and are potent modulators of the gut microflora through secretion of multiple antimicrobials including lysozyme (LYZ), phospholipase A2 group 1B (PLA2G1B), angiogenin ribonuclease A family member 5 (ANG5), and alpha-defensins (DEFAs), amongst others [15]. Multiple allelic variants of *NOD2*, *ATG16L1*, and *XBP1*, are associated with ileal Crohn’s disease [16–19] and have been identified in animal models to cause clear PC phenotypes, including lower DEFA expression [20], defects in autophagy, granule formation, and secretion [19, 21], and uncompensated ER stress [22]. While in vivo models currently provide the most physiologically representative system to probe PC biology, they are inherently complex and poorly scaled, hindering basic research into molecular mechanisms of disease and the potential for scalable therapeutic screening. Recently, conventional intestinal organoids were used to describe the dynamics of PC degranulation in response to multiple agonists [23] and to assess PC suppression of enteric pathogens [24]. While these organoid studies are arguably more representative than other in vitro systems, the question of physiological fidelity of this heterogeneous system remains unanswered, especially given that the timescales to derive conventional organoids is typically less than a week, while in vivo the lifespan of a PC is on the order of several weeks.

To improve the representation of specific cell types in intestinal organoids, investigators have utilized cellular engineering approaches starting with ISCs to derive multiple enriched or specialized models. These include enterocytes with improved intestinal ion transport [25], epithelial monolayers capable of secretion and IgA transcytosis [26], and organoids enriched for the rare secretory EEC population [27]. However, in each instance, there has been no global comparison of the extent to which intestinal organoids, or further specialized derivatives, recapitulate defined in vivo cell types and states. Moving beyond the generation of in vivo tissue maps towards mechanistic insights, particularly in disease settings, will require an understanding of how the in vitro organoid models utilized for such studies represent the cell types and states identified beyond single marker genes.

Here, we provide a global comparison between the in vivo cell states of the murine small intestinal epithelium and the in vitro conventional intestinal organoid, and establish a systematic workflow for improving the physiological representation of stem cell-derived cell states to enable the creation of high-fidelity in vitro models. Taking the PC as a test case, we utilize massively parallel single-cell transcriptomics (scRNA-seq; Seq-Well) [28] to benchmark the conventional organoid model against its in vivo counterpart and identify differences in developmental pathway signaling between in vitro and in vivo cell states. Single-cell transcriptomic approaches were key in enabling this study as epithelial cell types are challenging to reliably and prospectively isolate by fluorescence-activated cell sorting (FACS) due to the absence of robust surface markers and the spectrum of differentiation states present. This profiling guides the rational augmentation of signaling pathway activity during stem cell differentiation with a small molecule chemical induction method we previously validated to enhance global *Lyz* gene expression [29]. We validate our approach by generating an enhanced in vitro physiological mimic of the in vivo PC and provide a detailed characterization of the derived cell state through morphologic, proteomic, transcriptomic, and functional assays based on known signatures of in vivo PCs. Furthermore, we use our enhanced model and findings from its transcriptomic and proteomic characterization to identify *Nupr1* as a potential stress-response factor that facilitates the survival of PCs, demonstrating the improved ability to examine gene function in vitro within a more representative cell type.

## Results

### Using the PC to benchmark cell type representation of conventional organoids against their in vivo counterparts

**Fig. 1:**
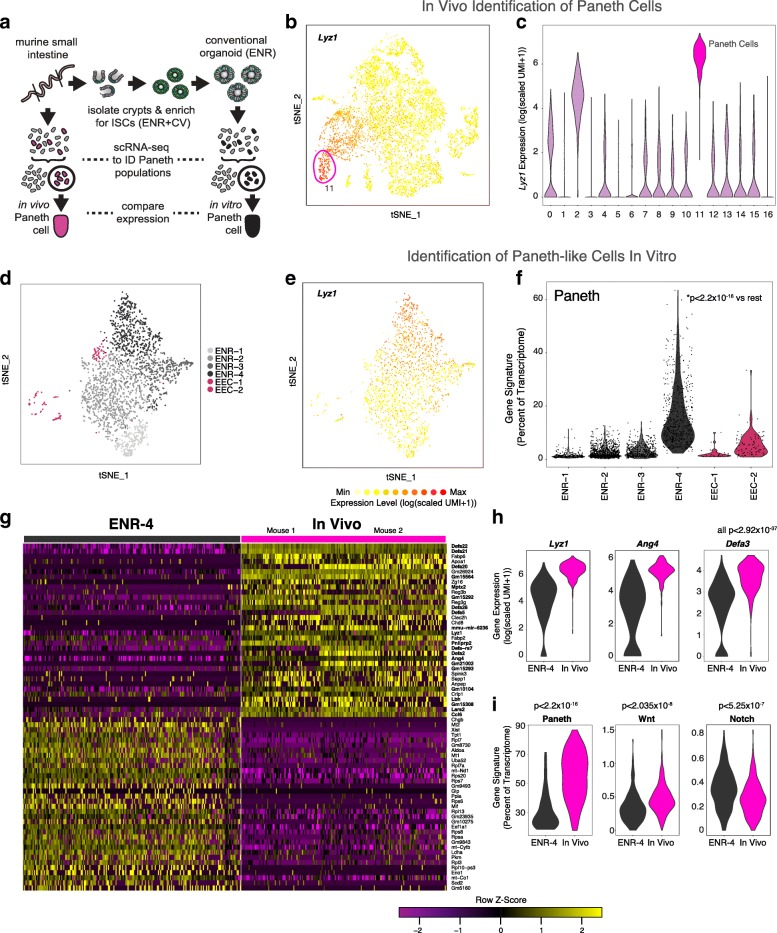
Transcriptional benchmarking of in vitro Paneth cells (PCs) to in vivo. **a** Schematic of intestinal epithelial cell isolation from terminal ileum for unbiased identification of in vivo PC signature genes, and system for intestinal stem cell (ISC) enrichment to characterize in vitro PCs, via high-throughput scRNA-seq. **b** Marker gene overlay for binned count-based expression level (log(scaled UMI + 1)) of *Lyz1*, a canonical PC marker gene, on a tSNE (t-stochastic neighbor embedding) plot of 7667 small intestinal epithelial cells isolated from the terminal ileum; receiver operating characteristic (ROC)-test area under the curve (AUC) = 0.995, *n* = 2 mice, independent experiments (Additional file 1: Table S1). **c** Violin plot for the count-based expression level (log(scaled UMI + 1)) of *Lyz1* across clusters identified through shared nearest neighbor (SNN) analysis (see Methods) over small intestinal epithelial cells; *n* = 196 cells in cluster 11, 7667 cells in total. **d** A tSNE plot of 2513 cells, with clusters identified through SNN (Additional file 1: Table S1 for full gene lists with ROC-test AUC > 0.60) from conventional ENR organoids; *n* = 6 wells of ENR organoids. **e** Marker gene overlay for binned count-based expression level (log(scaled UMI + 1)) of *Lyz1* on a tSNE plot from; ROC-test AUC = 0.856. **f** Violin plot of expression contribution to a cell’s transcriptome of PC genes across ENR organoid clusters from (**d**) (In vivo PC gene list AUC > 0.65, Additional file 1: Table S1); effect size 0.721, ENR-4 vs. all ENR, *t test *p* < 2.2 × 10^−16^. **g** Row-normalized heatmap of top differentially expressed genes using bimodal test over single-cells from the top 200 PC-like cells from ENR-4 and the 196 in vivo PCs (cluster 11, from (**c**)); *bimodal test, all displayed genes *p* < 1.89 × 10^−16^ or less with Bonferroni correction. **h** Violin plots for the count-based expression level (log(scaled UMI + 1)) of *Lyz1*, *Ang4*, and *Defa3* in ENR and in vivo PCs; *bimodal test, all *p* < 2.92 × 10^−37^ or less with Bonferroni correction. **i** Violin plot of expression contribution to a cell’s transcriptome of PC genes (effect size 1.25, InVivo vs. ENR, *t test *p* < 2.2 × 10^−16^), Wnt pathway (effect size 0.559, InVivo vs. ENR, *t test *p* < 2.035 × 10^−8^) and Notch pathway (effect size −0.500, InVivo vs. ENR, *t test *p* < 5.25 × 10^−7^) genes (see Additional file 2: Table S2 for gene lists)

Conventional intestinal organoids produced from the spontaneous differentiation of ISCs have been used to study PCs in vitro in multiple contexts [23, 24]. These in vitro PCs exist as part of a heterogeneous system, yet to be rigorously benchmarked against their in vivo counterparts. To better understand the composition of PCs within conventional organoids and how well those PCs approximate their in vivo counterparts, we sought to globally compare the conventional organoid-derived PCs and their in vivo counterparts through a single-cell transcriptomic approach (Fig. 1a).

To relate the organoid-derived PC state to in vivo PCs, we first generated an unbiased reference in vivo scRNA-seq data set. We performed massively parallel scRNA-seq using the recently developed Seq-Well platform [28] on epithelial cells from the ileal region of the small intestine acquired as two biological replicates (see Methods). We assessed quality metrics for the number of genes, unique molecular identifiers (UMIs), mitochondrial genes, and ribosomal genes, all of which fell within expectations (all cells average: 1043 genes, 2168 UMIs, 5.4% ribosomal genes, 10.4% mitochondrial genes). UMI-collapsed cell-by-gene (7667 cells × 17,505 genes) expression matrices were analyzed using Seurat (see Methods), performing dimensionality reduction, graph-based clustering, and deriving lists of cluster-specific genes in order to identify PCs. Within the spectrum of cell types, we identified two clusters (2 and 11) enriched for *Lyz1* expression (Fig. 1b, c), of which we determined cluster 11 to be fully mature PCs (*n* = 189 cells) based on uniform expression of a set of associated antimicrobial peptide marker genes such as *Defa22*, *Defa21*, and *Ang4* (receiver operating characteristic (ROC) test, area under the curve (AUC) > 0.99 for markers listed; cluster 11 average: 866 genes, 3357 UMI, 3.5% ribosomal genes, 4.8% mitochondrial genes) (Additional file 1: Table S1). We further utilized these genes (genes with AUC > 0.65 for in vivo PC) throughout our study to relate organoid-derived cell states to in vivo PCs. They are fully inclusive of the 14 high confidence markers described for Paneth cells from the terminal ileum in the recently published mouse small intestinal atlas [3]. Of note, we extended our gene list beyond truly specific marker genes that are not expressed in other cell types as we were interested in a more comprehensive set of PC-enriched genes for further comparison.

We next performed scRNA-seq using Seq-Well on conventional organoids derived from a single donor ISC-enriched state (Fig. 1a). Beginning with murine small intestinal crypts, we directly enriched for LGR5+ ISCs over 6 days following isolation within a Matrigel scaffold and medium containing recombinant growth factors EGF (E), Noggin (N), and R-spondin 1 (R), small molecules CHIR99021 (C), and valproic acid (V), as well as Y-27632 for the first 2 days to inhibit rho kinase and mitigate anoikis, as previously described (ENR+CV) [29]. To ensure reproducibility within our system and limit the risk of interference in our chemical induction approach, we conducted our study exclusively with recombinant growth factors and not cell line-derived conditioned media. Cells were passaged into conventional ENR culture for an additional 6 days to allow multi-lineage differentiation and produce stem cell-derived in vitro PCs. Following scRNA-seq, we computationally identified six clusters (amongst 2513 cells × 16,198 genes meeting quality standards, see Methods) in ENR organoids, which we label as ENR1-4, and EEC-1 and -2 for two EEC types (Fig. 1d). We identified ENR-4 as the cluster most enriched for *Lyz1* and our PC reference gene set (effect size 0.721, ENR-4 vs. all ENR, *t test *p* < 2.2 × 10^−16^; for effect size details see Methods) (Fig. 1e, f). Having identified ENR-4 as the cell state of interest in organoids, we directly compared the top 200 most PC-like cells in ENR-4 to in vivo PCs by performing differential expression analysis (Fig. 1g). In comparing the two cell types, it became evident that the majority of genes enriched by in vivo PCs were defensins and antimicrobials, including *Defa22*, *Defa21*, *Zg16*, *Ang4*, *Defa3*, and *Lyz1* (all *p* < 2.92 × 10^−37^, bimodal test, Bonferroni corrected for multiple comparisons) (Fig. 1g, h). ENR-4 cells were enriched for *Chgb*, an enteroendocrine marker, and translational biosynthetic genes likely indicative of the high rates of proliferation present in ENR organoids (Fig. 1g). We further note the difference in genes arising from non-sex matched comparison, like *Xist*, as a limitation of our comparison between a single donor for organoid derivation. Beyond these selected genes, we note a global reduction in the fraction of the transcriptome of ENR-4 cells producing the total cadre of in vivo PC marker genes (effect size 1.25, InVivo vs. ENR, *t test *p* < 2.2 × 10^−16^), suggesting that the current in vitro organoid-derived PCs are suboptimal for physiological studies (Fig. 1i).

Modulating key developmental pathways of stem cell-derived systems has emerged as a paradigm in bioengineering to rationally generate cell types for basic research and therapeutic aims [30, 31]. Specifically, modulating Wnt and Notch signaling has been suggested in the literature to increase the frequency and magnitude of *Lyz1* expression and protein in ISC-derived cells [29, 32–34]. Leveraging the single-cell transcriptomes of our in vitro- and in vivo-derived PCs, we confirmed that Wnt target genes are enriched in vivo relative to in vitro PCs (effect size 0.559, InVivo vs. ENR, *t test *p* < 2.035 × 10^−8^) and Notch target genes were decreased (effect size −0.500, InVivo vs. ENR, *t test *p* < 5.25 × 10^−7^) (Fig. 1i, Additional file 2: Table S2). As a result, we sought to comprehensively test if driving Wnt and inhibiting Notch truly results in a more physiologically representative PC versus the organoid-derived PC, beyond bulk measures of increased *Lyz1* expression.

### Rationally guided chemical induction of Wnt and inhibition of Notch drives PC marker enrichment

**Fig. 2:**
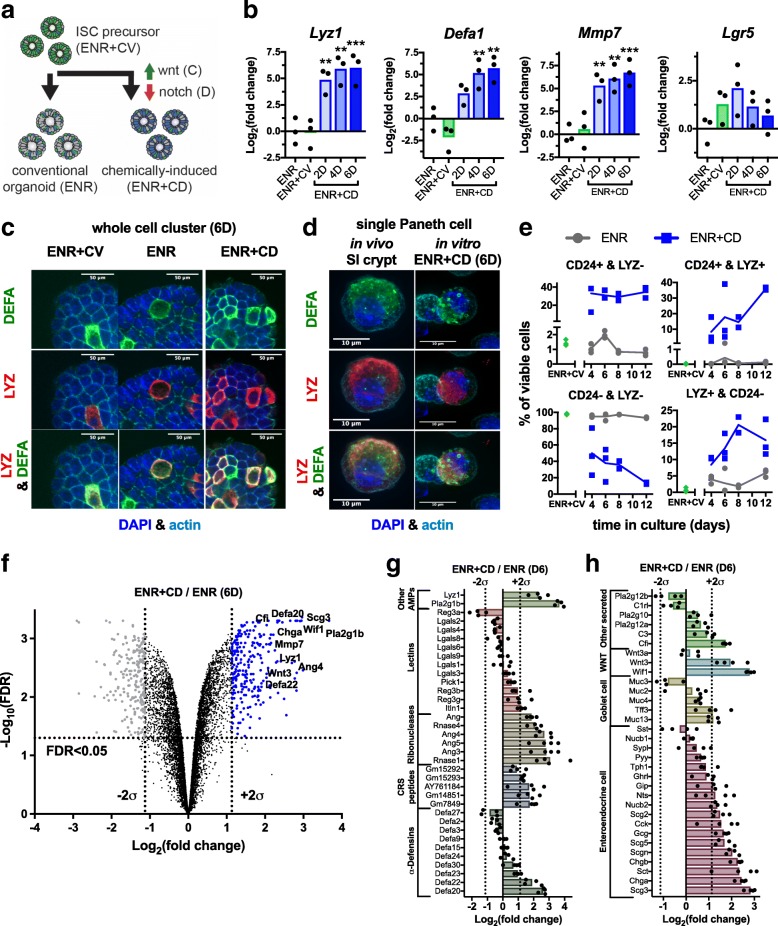
Establishing chemically induced Paneth cell (PC)-enriched cultures. **a** Schematic of small molecule-driven differentiation of LGR5+ ISCs (C - CHIR99021, D - DAPT) and non-specific differentiation. **b** mRNA expression of PC (*Lyz1*, *Defa1*, *Mmp7*) and ISC (*Lgr5*) markers relative to ENR, for ENR+CV and ENR + CD at 2 (D2), 4 (D4), and 6 days (D6) (*n* = 3 biological replicates; two-way ANOVA with multiple comparison test vs. ENR; ** adj. *p* < 0.01, *** adj. *p* < 0.001). **c** Representative confocal imaging of whole cell clusters for PC antimicrobials following 6 days in ENR+CD versus ENR and ENR+CV: stained for anti-DEFA, anti-LYZ and counterstained with DAPI and for actin (phalloidin). **d** High-resolution fluorescent imaging of in vivo and in vitro single cells from 6-day culture in ENR + CD shows similar morphology and antimicrobial expression: stained for DEFA and LYZ, and counterstained with DAPI and for actin (phalloidin). **e** Viable cell populations from ENR, ENR+CD, and ENR+CV precursor culture have distinct populations based on CD24 and LYZ content, indicative of PC maturity (*n* = 3 biological replicates; ENR + CV, days 4, 6, 12, *n* = 2 biological replicates day 8). **f** Volcano plot of differentially regulated proteins between 6-day (6D) ENR + CD and ENR cells shows clear enrichment in secreted and PC-associated proteins (labeled). Cut-offs are 2 standard deviations outside the mean expression level of the set and FDR < 0.05. **g** Rank-order log fold change of detected PC antimicrobial proteins and between 6-day ENR + CD and ENR cultures (*n* = 4). **h** Rank-order log fold change of detected secretory proteins associated with EEC and goblet lineages in ENR + CD relative to ENR cultures (*n* = 4)

Beginning with an LGR5+ ISC-enriched population (ENR+CV), we sought to profile how the modulation of Wnt and Notch signaling through small molecule inhibitors would alter the in vitro PC state, as suggested by our transcriptional profiling. We performed chemical induction (CI) using the previously identified compounds C to drive Wnt signaling and DAPT (D), a gamma-secretase inhibitor, to inhibit Notch (ENR+CD) (Fig. 2a) and measured bulk gene expression of PC (*Lyz1*, *Defa1*, *Mmp7*) and ISC (*Lgr5*) markers every 2 days for a total of 6 days (Fig. 2b). ENR+CD-treated cells had statistically significant increases in *Lyz1* (adj. *p* = 0.005, see Methods) and *Mmp7* (adj. *p* = 0.005) within 2 days compared to ENR, with differences plateauing around 4 days. *Defa1* (adj. *p* = 0.004) expression was significantly increased by day 4 and plateaued by day 6 in ENR+CD versus ENR populations. *Lgr5* expression in ENR+CD at 2 days versus ENR showed an insignificant plateau of expression, which trended down by 6 days. This may be indicative of an expansion in ‘label-retaining’ secretory precursors [35]. The precursor population ENR + CV had no significant difference in PC or ISC markers relative to ENR. The significant increase in PC gene expression in ENR + CD relative to ENR and ENR+CV over the 6-day treatment suggests rapid enrichment following CI, supporting our hypothesis that alterations in Wnt and Notch result in superior PC enrichment in vitro.

To phenotypically describe PC enrichment following CI, we performed imaging and immunocytochemistry for PC-associated features. After 6 days of ENR + CD, cell populations exhibited darkened annular morphology consistent with increased numbers of granule-rich cells (Additional file 3: Figure S1A). Confocal microscopy of whole cell clusters stained for anti-DEFA and anti-LYZ showed an increase in LYZ+ and DEFA+ cells in ENR + CD compared to both ENR and ENR + CV (Fig. 2c). Single-cell counting of confocal imaging showed a significant increase of DEFA and LYZ co-staining cells in ENR + CD (20–30% of cells) versus either ENR or ENR + CV (both < 5%; adj. *p* = 0.0001) (Additional file 3: Figure S1B). Additionally, normalized z-axis profiles of individual co-staining cells within cell clusters revealed a consistent distribution of DEFA (luminally polarized) and LYZ (diffuse) (Additional file 3: Figure S1C 1–3). High-resolution fluorescent imaging of individual co-staining cells from freshly isolated small intestinal crypts (in vivo equivalent) and 6-day ENR + CD-treated cells showed a similar polarized distribution of LYZ- and DEFA-stained granules, although freshly isolated cells appeared to be more granular than CI-PCs (Fig. 2d).

To confirm the extent of enrichment seen in whole population imaging, the prevalence of PCs in ENR + CD relative to ENR was assessed by flow cytometry over the course of 12 days, a longer term culture than typical for conventional organoids. We identified an in vivo PC phenotype as CD24 and LYZ co-positive cells, as per previous reports [36], and noted the presence of single-positive LYZ+ or single-positive CD24+ populations, indicative of alternative cell differentiation, immature, or non-physiological PCs (representative populations Additional file 3: Figure S1D, representative gating Additional file 3: Figure S1E). ENR + CD had substantial enrichment at all time points for double-positive, and single-positive LYZ+ or CD24+ populations relative to ENR, as well as a consistent decrease in the double negative population in agreement with the PC phenotype (Fig. 2e). Notably, both ENR and ENR + CD experience declines in total cell viability, with ENR + CD having greater survival at longer times, suggesting both a reduction in anoikis, a potentially physiological ‘long-lived’ PC phenotype in ENR + CD versus ENR, or an enhancement in niche-supporting functionality (Additional file 3: Figure S1F). Overall, imaging and flow cytometry demonstrate a significant increase in cells morphologically resembling in vivo PCs with respect to granularity, polarity, and antimicrobial co-expression in ENR + CD compared to conventional ENR organoids (Fig. 2c–e and Additional file 3: Figure S1A–F).

### Chemically induced PC proteome is enriched for components of secretory lineages

With ENR + CD apparently providing a more prevalent and physiological PC population, we sought to more globally characterize the differences between in vitro PCs (ENR vs. ENR + CD) at 6 days. Because our single cell transcriptomic comparison revealed that many of the differential genes between PCs in conventional organoids and in vivo were lineage-defining protein products, we sought to assess the total intracellular proteome between the conventional organoid and our chemically induced model through liquid chromatography mass spectrometry (LC-MS/MS)-based proteomics. We quantified relative protein abundance using isobaric mass tag labeling from four ENR and four ENR + CD samples and analyzed them in a single 10-plex by LC-MS/MS (Additional file 4: Figure S2A). We identified 8015 unique proteins within all samples; each replicate pair (ENR + CD/ENR) was normally distributed (not shown) and correlated with all others, indicating consistent proteome enrichment (Additional file 4: Figure S2B). We looked at the sample pairs in aggregate and classified proteins significantly enriched in ENR + CD and ENR by a false discovery rate (FDR) < 0.05 and log fold change (± 2*σ*) (Fig. 2f and Additional file 5: Table S3). There were 249 ENR + CD-enriched proteins, 212 ENR-enriched proteins, and 7553 shared proteins. Known PC markers, including LYZ, DEFAs, and other secretory pathway components, were identified as significantly enriched in ENR + CD versus ENR alone. Of known antimicrobial proteins produced by PCs, we detected 10 DEFAs, 5 CRS peptides, 6 ribonucleases, 12 lectins, LYZ1, and PLA2G1B with differential abundance between ENR + CD and ENR (Fig. 2g). Each class of antimicrobials had at least one ENR + CD enriched protein (+ 2*σ*), with the ribonucleases significantly enriched and a majority of the lectins and DEFAs unregulated between the two conditions. Proteins associated with the EEC lineage (secretogranins, chromogranins, and neuropeptides) were also enriched in ENR + CD, in addition to multiple other secreted components, including Wnt ligands, and the complement pathway components C3 and CFI (Fig. 2h). In sum, we see a broad diversity of PC-associated antimicrobials with some enrichment of EEC-associated proteins in ENR + CD relative to ENR.

Additionally, we characterized enriched biological functions, cellular compartments, and molecular functions using DAVID v6.8 and the gene ontology database. All sets had high database coverage (greater than 85%) of queried proteins. The ENR + CD proteome is significantly enriched for extracellular and protein processing compartments and secretory-associated functions (Additional file 4: Figure S2C), while the ENR proteome favors translation, intracellular compartments, and translational activities (Additional file 4: Figure S2D). Of note, there are the extracellular exosome and calcium ion-binding associated proteins in the ENR + CD proteome that are indicative of the intestinal epithelial secretory phenotype (for a complete list of DAVID enrichments, refer to Additional file 6: Table S4). These functional enrichments further support the notion that the ENR + CD-cultured organoids are enriched in secretory cells, including PCs, although it does not rule out potential co-enrichment for the EEC lineage. Finally, we sought to identify transcription factors (TFs) that may mediate PC-specific differentiation using GSEA [37, 38] with the MSigDB transcription factor target (v5.2) gene set database [39] with a moderately conservative cutoff (see Methods). We generated an enrichment map [40, 41] of several TF targets significantly enriched in both the ENR + CD and ENR proteomes. In ENR + CD, the nuclear receptors for progesterone, aldosterone, and glucocorticoid, as well as the cellular differentiation-implicated TAL1, RP58, and NRSF, were significantly enriched. In ENR, the primary known enrichment was for the cell cycle and proliferation-related E2F TF family (Additional file 4: Figure S2E). These potential TFs are consistent with CI-PC treatment driving expected terminal differentiation of specialized cells, as opposed to conventional organoid culture, which supports a broad mix of intestinal epithelial cells, including proliferating populations. Furthermore, this analysis suggests potential targets, such as progesterone, aldosterone, and glucocorticoid, to modulate the differentiation programs of this secretory cell population in future studies.

### Single-cell RNA sequencing profiles heterogeneity of chemically induced PCs, revealing subsets with improved transcriptional similarity to in vivo

**Fig. 3:**
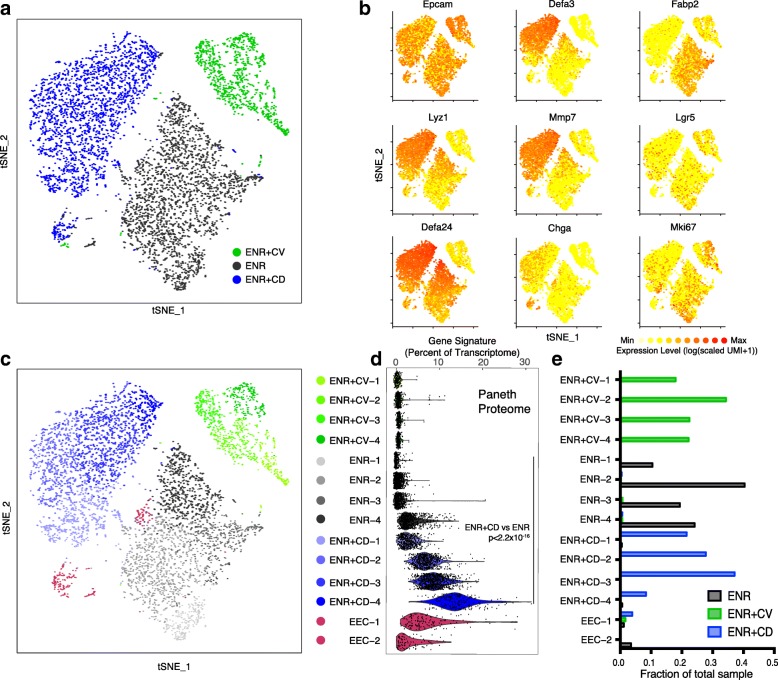
Single-cell RNA-sequencing reveals cellular composition across treatments and origins of proteomic data. **a** A tSNE plot of single cells derived from ENR + CV (*n* = 985 cells), ENR (*n* = 2544 cells), and ENR + CD (*n* = 2382 cells) harvested at day 6 of differentiation, colored by treatment; *n* = 6 wells for each condition. **b** Marker gene overlays (on plot from (**a**)) for binned count-based expression level (log(scaled UMI + 1)) of individual genes of interest. **c** A tSNE plot, with clusters identified through SNN graph-based clustering (see Additional file 1: Table S1 for marker gene lists), highlighting distinct cell states within each organoid; opacity of density clouds correspond to the Paneth cell score of ENR-4, ENR + CD-3, and ENR + CD-4 clusters (see Fig. 4b). **d** Violin plot of expression contribution to a cell’s transcriptome of ENR + CD proteome-enriched genes across organoid clusters from (**c**) (see Additional file 1: Table S1 for full gene list); effect size 2.40 ENR + CD-4 vs. all cells, *p* < 2.2 × 10^−16^. **e** Frequency of each cluster observed within each organoid condition as a fraction of the total cells in each condition

With the apparent co-enrichment of canonical PC and EEC proteins in the ENR + CD proteome, we sought to identify whether we produce a homogenous population of mixed-lineage secretory cells or a spectrum of unique cell states between EEC and PC. We performed scRNA-seq using the Seq-Well platform on cells from ENR + CD and the precursor ENR + CV conditions to analyze alongside conventional ENR organoids. To ensure experimental robustness, we assessed quality metrics for the number of genes, UMIs, mitochondrial genes, and ribosomal genes by cluster, all of which fell within expectation (Additional file 7: Figure S3). UMI-collapsed digital gene expression matrices were analyzed using Seurat (see Methods), and displaying all three treatments (ENR + CV, ENR, ENR + CD) in tSNE space demonstrated clear separation between each condition (Fig. 3a), illustrating that the unique transcriptional differences induced by each treatment were conserved across all cells. Plotting key genes demonstrated that, as expected, all cells expressed high levels of *Epcam*, that ENR + CV cells had enhanced *Mki67*, a marker of proliferation, that the ENR + CD condition led to enrichment of cells expressing antimicrobial *Lyz1*, *Defa24*, *Defa3*, *Mmp7*, and EEC marker *Chga*, and that ENR enriched for absorptive marker *Fabp2*-expressing cells (Fig. 3b).

To assess subpopulation structure and provide a more robust measure of composition beyond canonical marker genes, we performed unsupervised KNN graph-based clustering on the captured cells (Fig. 3c, d and Additional file 1: Table S1 for full gene lists), distinguishing four clusters in each treatment condition. We then scored individual clusters according to the amount of the transcriptome within each cell dedicated to synthesizing the respective enriched proteins from the bulk proteome data. We observed that ENR + CD clusters yield a significant enrichment for those proteins detected in the up-regulated proteome (effect size 1.38 ENR + CD vs. ENR clusters, *p* < 2.2 × 10^−16^) and that the down-regulated proteins were enriched in the ENR and ENR + CV conditions (Fig. 3d, e and data not shown). Intriguingly, at the level of clusters, the upregulated proteome was not evenly distributed across all cells in ENR + CD, but was most enriched in cluster ENR + CD-4, which represented approximately 10% of ENR + CD cells (effect size 2.40 ENR + CD-4 vs. all cells, *p* < 2.2 × 10^−16^) (Fig. 3d, e).

**Fig. 4:**
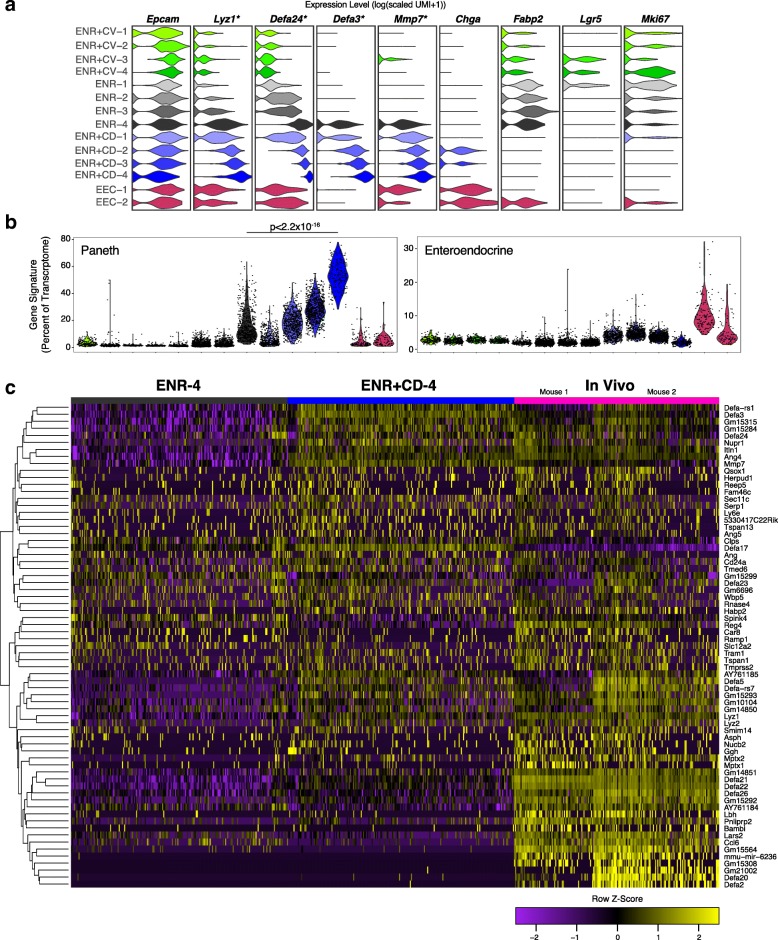
Transcriptional identity of chemically induced Paneth cells (CI-PCs) within conditions and related to in vivo PCs. **a** Violin plots for the count-based expression level (log(scaled UMI + 1)) of selected genes across called clusters, colors correspond to clusters in Fig. 4c; *t test, *p* < 6.80 × 10^−74^ or less with Bonferroni correction, for *Lyz1*, *Defa24*, *Defa3*, and *Mmp7* ENR + CD-4 relative to ENR-4. **b** Violin plot of expression contribution to a cell’s transcriptome of in vivo PC and enteroendocrine marker-cell genes (see Additional file 1: Table S1 for full gene list, AUC > 0.65); effect size 2.52 ENR + CD-4 vs. ENR-4, *p* < 2.2 × 10^−16^ for PC score; effect size 0.0465, *p* = 0.2339 ENR + CD-4 vs. ENR-4 for enteroendocrine cell score. **c** Row-clustered heatmap of z-scores (−2.5 to 2.5; purple to yellow) for defining genes (*n* = 69 with AUC > 0.65 of in vivo PCs, see Additional file 1: Table S1 for full gene list) across top 200 cells for PC score (Fig. 5b) from ENR-4 and ENR + CD-4 conditions compared to two biological replicates of in vivo PCs from the terminal ileum (*n* = 196 cells)

To address ENR + CD composition and how it relates to conventional organoids, we interrogated the expression of *Lyz1*, *Chga*, and other selected genes across each cluster (Fig. 4a). We noted that clusters ENR-4 and ENR + CD-4 shared expression of *Lyz1*, *Defa24*, *Defa3*, and *Mmp7*, yet ENR + CD-4 cells produced significantly more of each canonical PC gene (bimodal test, *p* < 6.80 × 10^−74^ for genes listed, Bonferroni corrected for multiple comparisons). Furthermore, both ENR-4 and ENR + CD-4 cells lacked expression of EEC genes like *Chga*, which was observed in the EEC-1 and EEC-2 clusters arising from mixed-grouping of the sample, as well as in ENR + CD-2 and ENR + CD-3 (Fig. 4a). Altogether, this suggests that ENR + CD drives PC differentiation while also inducing a secretory transition state (ENR + CD-2 and 3) expressing a mix of PC and EEC marker genes (Additional file 1: Table S1 for full gene lists).

We next sought to compare the states generated in vitro to those observed in vivo with our refined system*.* Using the gene list of in vivo PC markers and further defining a list for in vivo EECs (see Methods) captured on the Seq-Well platform (Additional file 1: Table S1), we observed that the percentage of a cell’s transcriptome dedicated to synthesizing defining Paneth genes was significantly enriched relative to ENR-4 in clusters ENR + CD-2, -3, and -4 (effect size 0.15, *p* < 3.43 × 10^−5^; effect size 0.829, *p* < 2.2 × 10^−16^; effect size 2.52, *p* < 2.2 × 10^−16^, respectively) with an increase in expression of EEC genes across ENR + CD-1, -2, and -3 but not ENR + CD-4 (effect size 1.30, *p* < 2.2 × 10^–16^; effect size 1.82, *p* < 2.2 × 10^–16^; effect size 1.118, *p* < 2.2 × 10^−16^; effect size 0.0465, *p* = 0.2339, respectively) (Fig. 4b). Notably, ENR + CD-4 cells (~10%) had a three-fold increase in the transcriptional resemblance to in vivo PCs relative to ENR-4 (53.4% of transcriptome ENR + CD-4 vs. 16.5% of transcriptome ENR-4) (quantification of Fig. 4b). Furthermore, 45% of ENR + CD cells express a secretory PC-like transcriptional phenotype that is at least two-fold enhanced relative to conventional organoids (33.9% of transcriptome ENR + CD-3 and -4 vs. 16.5% ENR-4). Comparing the ENR + CD-4 cells relative to in vivo PCs demonstrated a striking similarity relative to the difference observed between in vivo and ENR-4 cells (PC fraction of in vivo transcriptome: effect size 0.237 InVivo vs. ENR + CD-4, *p* < 0.0055; effect size 1.25 InVivo vs. ENR-4, *p* < 2.2 × 10^−16^ (Additional file 1: Table S1).

In Fig. 4c, we present a heatmap of scaled expression values for the top genes (AUC > 0.65) used for the in vivo Paneth score across ENR-4, ENR + CD-4, and the in vivo cluster used to define PCs. We observed that the enhanced PC phenotype in ENR + CD-4 (effect size 1.144 ENR + CD-4 vs. ENR-4, *p* < 2.2 × 10^−16^) correlated with a greater expression of signature genes, such as *Lyz1*, *Lyz2*, and *Defa5*, and a greater diversity of antimicrobial peptide genes, such as *Ang4*, *Defa3*, and the metalloprotease *Mmp7*.

To confirm and extend our findings of pathway-based modulation, we scored clusters for enrichment or depletion of canonical growth factor-induced pathways. CHIR99021 activates the Wnt pathway, and we observed a significant enrichment for Wnt target genes in all CI-PC clusters (effect size > 0.999, *p* < 2.2 × 10^−16^ for all ENR + CD clusters vs. ENR-4) (Additional file 8: Figure S4A). While DAPT is a Notch pathway inhibitor, levels of Notch target genes were largely greater than or equivalent to ENR-4 cells across CI-PC clusters, except for significant depletion in ENR + CD-4 (effect size −0.658, *p* < 2.2 × 10^−16^ ENR + CD-4 vs. ENR-4) (Additional file 8: Figure S4B). This suggests that complete Notch suppression is key for PC differentiation distinct from an EEC fate. Additionally, given the recognized role for distinct respiratory potential in enterocytes, ISCs, and PCs, we scored cells across respiratory electron transport genes [42, 43]. ENR + CD-4 had the lowest cluster score relative to all cell subsets (effect size −1.4649, *p* < 2.2 × 10^–16^) (Additional file 8: Figure S4C). Together, this suggests that Wnt signaling is necessary but not sufficient to specify the mature PC phenotype and that Notch and metabolic conditions play a larger role in the decision between PC and EEC fates.

### Chemically induced PCs mimic in vivo stimulant-induced secretion and demonstrate selective modulation of bacteria in co-culture

**Fig. 5:**
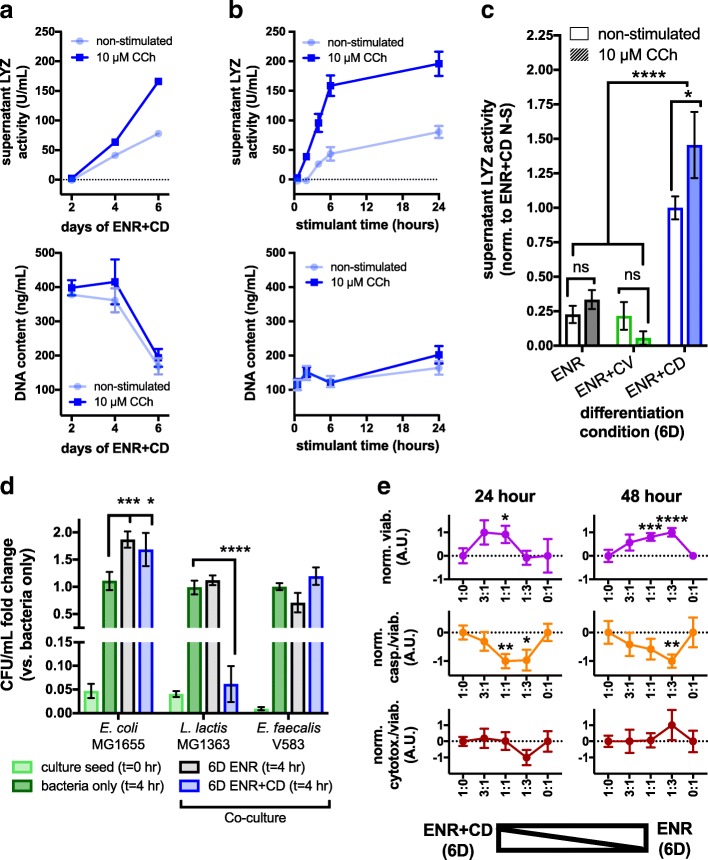
Chemically induced Paneth cells (CI-PCs) are functional in response to host and microbial stimuli. **a** Supernatant LYZ from 24-h basal and 10 μm CCh-stimulated LYZ cells at varying number of days in ENR + CD culture (top). DNA content from matched samples (bottom) (*n* = 8 well replicates; SEM error bars too small to visualize). **b** Supernatant LYZ from 6-day ENR + CD collected basally and following 10 μm CCh stimulation for 0.5, 2, 4, 6, and 24 h (top). DNA content from matched samples basally and following 10 μm CCh stimulation (bottom) (*n* = 8 well replicates). **c** 24-h basal (non-stimulated) and 10 μm CCh-stimulated LYZ secretion in 6-day ENR + CD versus ENR and ENR + CV (*n* = 8 well replicates; two-way ANOVA with multiple comparison test; ns non-significant, * adj. *p* < 0.05, **** adj. *p* < 0.0001). **d** 4-h co-culture of freshly passaged 6-day ENR and ENR + CD cells and select gram-negative and gram-positive aerobic bacteria (*n* = 13 well replicates; two-way ANOVA with multiple comparison test, * adj. *p* < 0.05, *** adj. *p* < 0.001, **** adj. *p* < 0.0001). **e** Normalized cellular viability, caspase activity per viable cell, and cytotoxicity per viable cell from 24-h and 48-h ENR and ENR + CD co-cultures at specified mixing ratios (*n* = 9 well replicates from three biological donors; one sample *t* test,* *p* < 0.05, ** *p* < 0.01, *** *p* < 0.001, **** *p* < 0.0001)

In addition to our morphological, proteomic, and transcriptional characterization of PC phenotype in ENR + CD and ENR, we sought to measure physiological function by assessing stimulant-induced secretion of antimicrobials. We assessed the dynamics of LYZ accumulation in the supernatant media of cultures following media wash, basally and after stimulation with carbachol (CCh), a cholinergic agonist known to induce PC secretion [44]. CCh (10 μM) induced a rapid accumulation of LYZ within 2 hours that plateaued around 6 hours post-wash (two-way ANOVA, stimulant *p* < 0.0001, time-point *p* < 0.0001) (Fig. 5b). The observed PC secretion in response to CCh is consistent with observations made in ex vivo crypts, though over appreciably longer time scales, likely due to the added diffusion barrier of the organoid structure and matrigel [44]. We next identified how LYZ secretion changes over the course of differentiation. Beginning with an ISC-enriched population, we assayed for secreted LYZ in cell culture supernatants every 2 days for 6 days of ENR + CD culture, following a 24-h stimulation with CCh or without (basal collection/non-stimulated). Notable increases in functional secretion (stimulated relative to basal) occurred at days 4 and 6 (two-way ANOVA, stimulant *p* < 0.0001, time-point *p* < 0.0001) (Fig. 5a). Compared to conventional organoids and ISC-enriched precursors, ENR + CD secreted significantly more basal LYZ (*p* < 0.0001) and was the only population that showed grossly measurable CCh-induced secretion (adj. *p* = 0.03) (Fig. 5c). This result is consistent with the observed enrichment between chemically induced populations relative to conventional.

Based on the broad spectrum of antimicrobials detected proteomically, transcriptionally, and functionally, we hypothesized that ENR + CD possess greater bactericidal effects than conventional organoids. We assayed for bacterial growth modulation by suspending cell clusters with common laboratory strains of gram-negative and gram-positive bacteria in exponential growth. CI-PCs significantly suppressed growth of gram-positive *L. lactis* MG1363 (adj. *p* = 0.0001), which did not occur with conventional organoids, indicative of increased PC-associated antimicrobial activity (Fig. 5d). Both ENR (adj. *p* = 0.0005) and ENR + CD (adj. *p* = 0.01) co-culture showed significant increase in gram-negative *E. coli* MG1655 growth but no appreciable effect was observed on the growth of gram-positive *E. faecalis* V583 versus bacteria alone (Fig. 5d). While this assay simplifies the PCs’ physiological environment and may not be a direct proxy for strain-specific growth modulation, it does demonstrate that the PC-enrichment of ENR + CD versus conventional organoids enables detectable in vitro bacteria species-specific PC antimicrobial response, opening avenues for future experimentation.

### Chemically induced PCs provide niche support and enhance conventional organoid survival

Beyond the generation of antimicrobial peptides, PCs provide niche support for ISCs. We sought to test if CI-PCs provided niche factors known to drive epithelial regenerative turnover. We performed co-culture experiments, mixing and re-plating cell populations derived from 6 days of ENR or ENR + CD culture and assayed co-culture viability, caspase activity, and cytotoxicity 24 and 48 h following re-plating in ENR media. If there was no appreciable interaction, positive or negative, between the two populations we would expect to see a linear trend of every measured variable throughout mixing ratios. However, we observe a significant positive interaction where the presence of both populations drives an overall increase in cellular viability, beginning at 24 h (one-sample *t* test 1:1 *p* = 0.037) and increasing at 48 h (one-sample *t* test 1:1 *p* = 0.001 and 1:3 *p* < 0.001) (Fig. 5e). This is likely due to a significant decrease in overall apoptosis relative to the total cell population (one-sample *t* test 24 h 1:1 *p* = 0.004 and 1:3 *p* = 0.032, 48 h 1:3 *p* = 0.003), and unrelated to changes in cellular cytotoxicity. We believe that the presence of a PC-enriched population (from ENR + CD) is driving this effect by providing increased soluble regenerative factors to the ISC population in ENR organoids, increasing the generation of new cells, and resulting in a lower overall rate of apoptosis.

### Mapping of in vivo PC-associated transcription factors to in vitro proteome and transcriptome reveals *Nupr1* as important in epithelial survival

**Fig. 6:**
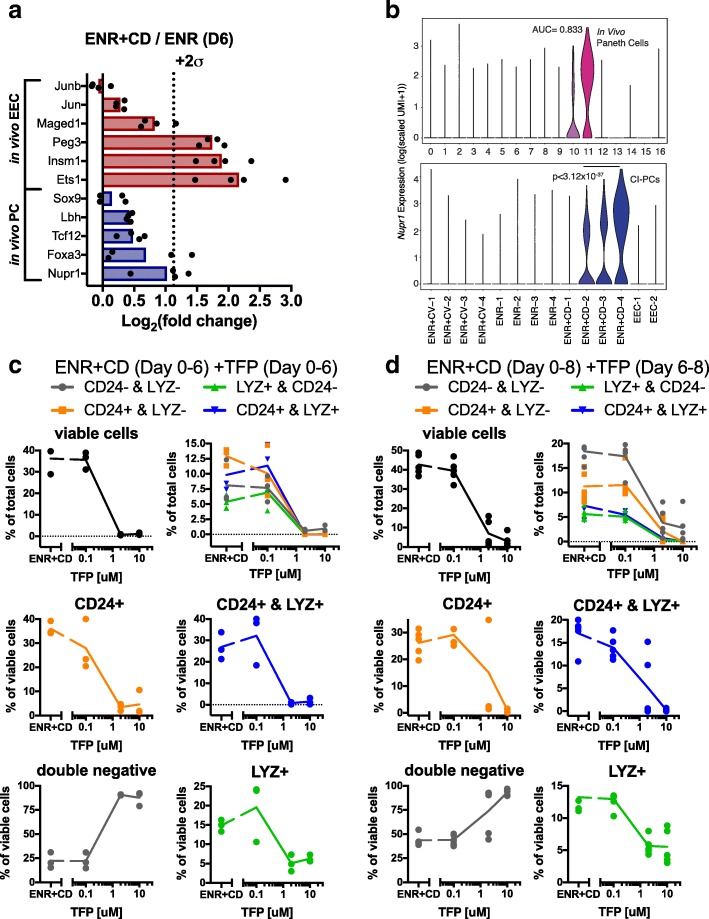
CI-PCs reveal putative function of *Nupr1* transcription factor in Paneth cell (PC) survival. **a** ENR + CD is enriched for in vivo PC and EEC transcription factors, including *Nupr1* (*n* = 4). **b** Violin plots for the count-based expression level (log(scaled UMI + 1)) of *Nupr1* across in vivo and in vitro called clusters. **c** *Nupr1* inhibition with trifluorperazine (TFP) treatment concurrent with 6-day ENR + CD differentiation reveals dose-dependent toxicity, with preference to PCs (CD24+ and LYZ+) and PC-like (CD24+ and LYZ+) populations as assessed by flow cytometry (*n* = 3 biological replicates). **d** 2-day TFP treatment following 6-day ENR + CD differentiation reveals dose-dependent toxicity, with preference to PCs (CD24+ and LYZ+) and PC-like (CD24+ and LYZ+) populations as assessed by flow cytometry (*n* = 5 biological replicates)

Finally, we sought to use this physiologically improved in vitro PC system (ENR + CD) to identify novel factors potentially supportive of PC survival or differentiation. Using our in vivo PC and EEC gene lists, and filtering for only TFs (using TFdb, downloaded September 2017) [45], we identified a set of PC- or EEC-specific TFs. We mapped these TFs to our in vitro proteome (Fig. 6a and Additional file 5: Table S3), which revealed the previously unreported NUPR1 as the most enriched PC-specific TF in ENR + CD. This finding was supported by differential expression between ENR + CD-2 (most EEC-like cells) and ENR + CD-4 (*p* < 3.12 × 10^−37^, bimodal test, Bonferroni corrected for multiple comparisons) (Fig. 6b). We further identified *Nupr1* in our in vivo PC populations, which showed specific and enriched expression of *Nupr1* by in vivo PCs (ROC test, AUC = 0.833) (Fig. 6b). Intriguingly, *Nupr1* is a stress-response gene, known to promote cellular survival and senescence through mediation of autophagy, and has primarily been studied in the context of cancer [46–48]. Autophagy and stress response have repeatedly been implicated through GWAS study in PCs in IBD; however, *Nupr1* has only ever been reported in a single IBD GWAS study, and its role in PC biology has not been formally investigated [49]. With our model, we sought to test the role of NUPR1 on in vitro PC survival through the small molecule inhibition of NUPR1 with trifluoperazine (TFP) [50, 51]. While genetic perturbation may provide for more specific effect measurement, we chose to use TFP as a simple, albeit less specific, modulator, as the complexity involved in selecting for a genetically perturbed population of PCs in organoids, if *Nupr1* is a survival gene, is beyond the scope of the present study. We first tested how different dosages impact PC differentiation in combination with ENR + CD for 6 days, where doses above 1 μM led to near total cell death, and where the few surviving cells were primarily non-PC (Fig. 6c). This suggests that *Nupr1* is likely critical to cellular survival during the CI differentiation process. We also tested the addition of TFP for 2 days following a 6-day course of ENR + CD, where again a profound, but not total, decline in cellular viability was observed. Further, it appears that TFP treatment is selectively more toxic to PC and PC-progenitor populations relative to non-PC populations (Fig. 6d). In total, this initial investigation suggests that NUPR1 may be a critical TF in PC development and survival, which carries therapeutic implications which we will seek to validate with expanded gene-perturbation studies in vitro and in vivo in future work.

## Discussion

We sought to directly compare a specific cell type present in vivo to that derived in an intestinal organoid in vitro, with the main goal of understanding the nature and extent of divergence between the in vitro and in vivo conditions. Empowered by recent advances in massively-parallel scRNA-seq, we employed a generalizable approach to define the relation between in vivo and organoid-derived in vitro PCs and employ a rationally identified intervention to improve in vitro representation through chemical modulation of developmental pathways. Our scRNA-seq approach enabled the identification of populations of interest both in vivo and in vitro and the analysis of differences between subpopulations which would have otherwise been greatly obscured in a bulk analysis of this heterogeneous system. While others have recently used scRNA-seq to profile the heterogeneity of intestinal organoids [4] and the murine small intestinal epithelium [3], we provide a direct comparison between the model and system. This comparison is key to understanding what complex models, such as intestinal organoids, really represent, how they may best be utilized and rational strategies for improvement.

In our comparison, we identified that the PC-state of conventional intestinal organoids poorly represents the extent and breadth of antimicrobial gene expression, and that modulation of Wnt and Notch during differentiation may improve physiological representation. Using a combination of small molecule promotion of Wnt and inhibition of Notch signaling that had previously been shown to improve the bulk expression of PC-marker gene *Lyz1* [29], we drove a secretory differentiation program and enriched for mature PCs with greater diversity and expression of antimicrobial peptides relative to existing in vitro models and, thus, are more representative of in vivo PCs. Imaging of this population revealed that they are positive for the antimicrobials LYZ and DEFA, clearly polarized, and granule rich, suggestive of a mature PC. This population is approximately six-fold more abundant in ENR + CD than an ENR organoid, as confirmed through image quantification, flow cytometry and scRNA-seq. We further characterized the subpopulation enrichments of our ENR + CD culture and directly compared it to conventional organoids. We identified two subpopulations in scRNA-seq (ENR + CD-3 and ENR + CD-4) that account for approximately half of the ENR + CD-treated cells with a high-degree of transcriptional similarity to in vivo PCs, a greater percentage/matching than the ENR-subpopulation that most resembles an in vivo PC (ENR-4). From this analysis, we believe that in vitro PCs characterized in the past [23, 24] likely represent secretory precursor populations lacking the full phenotypic repertoire of the in vivo PC, which we identify as the approximately 5% of single-staining LYZ+ cells present in ENR organoids as assessed by flow cytometry (Fig. 2e). This finding makes sense, given that conventional organoid culture often occurs on the time scale of a week, while in vivo PCs are relatively long lived (several weeks), develop in non-sterile conditions, and presumably would require longer periods of culture to reach maturity in vitro. Indeed, our ENR + CD cultures show increasing PC populations up to 12 days of culture and may likely continue gaining at longer time points. While our approach moves us much closer to generating the in vivo PC (fraction of in vivo transcriptome: effect size 0.237 InVivo vs. ENR + CD-4, *p* < 0.0055; effect size 1.25 InVivo vs. ENR, *p* < 2.2 × 10^−16^), we still do not capture the total amount of antimicrobial peptides present in vivo, and propose pathways to modulate in future studies.

Evidence suggests that PC antimicrobial expression and function are influenced by genetic background [52] and implicated in intestinal disease, including IBD [53]. How genetic background may influence differentiation through this protocol is yet to be studied but especially prudent, as we demonstrated the ability to detect a broad spectrum of antimicrobial proteins and peptides and their differential abundance within a PC-enriched population. Interestingly, we identified that the same subpopulation (ENR + CD-4) with the most transcriptional overlap to our bulk ENR + CD-enriched proteome also most closely resembles the in vivo PC. While this subpopulation does not account for the majority of ENR + CD-cultured cells, it appears that ENR + CD-4 consistently drives the PC phenotype in vitro. In addition to assessing the role of genetic background or disease state on antimicrobial content, our platform also affords the ability to interrogate how alterations in protein processing and storage in PCs affects the proteome, which has been shown to drive shifts in the microbiome and may be implicated in disease [54, 55]. Finally, while we demonstrate an enriched phenotypic spectrum of antimicrobials and Wnt ligands, we also identified several neuropeptides and hormone products associated with the EEC lineage within our system. Given that multiple studies have linked the differentiation of PCs and EECs through a common progenitor population [56], it is reasonable to expect enrichment in one population would also allow for some overlap with the other, as we see in our scRNA-seq data. Future experiments will seek to leverage these transition states to more formally identify genetic programs that underlie common or unique developmental trajectories.

To understand how our chemical induction led to distinct secretory subpopulations within the CI-PCs, we mapped Wnt, Notch, and metabolic gene sets onto each subpopulation. In our system, Notch-signature is highest in the stem cells and EECs, lower in enterocytes, and lowest in PCs. Our system’s Wnt signature is relatively decreased in enterocytes (ENR largely) and increased in PCs and EECs, both of which occur predominantly in the Wnt-driven condition ENR + CD (CI-PCs). In total, this suggests that Wnt is necessary for ISCs to commit to PC and EEC lineages and that future experimentation with specific synthetic Wnt ligands [57] may prove fruitful in distinguishing Wnt target genes that discriminatorily yield PCs or EECs. Additionally, it is clear that strong Notch inhibition is important for mature PC development, possibly as a balance between differentiation and cell survival. Interestingly, we also see a notable gradient in cellular respiration across subpopulations, lowest in the PC and highest in the stem cell and EEC lineages, in agreement with recent work on the metabolic differences within the stem cell niche [43], as another potential cue to further specify PC differentiation. Future studies should incorporate temporal aspects to growth factor delivery akin to what has been shown for degradable matrices [58] to enhance purity and yield, and explore the role of metabolic utilization in addition to growth factor signaling in cellular fate determination. Indeed, while we implemented an approach of chemical induction to drive model improvement, there are many other approaches which may be implemented to seek similar shifts in the composition of organoid systems, such as using novel ligands or tuning of the material supports. Further, we appreciate that many investigators have begun using R-spondin or Wnt conditioned medias in their intestinal organoid cultures; however, to ensure consistency and control of the system upon chemical induction we chose to use recombinant growth factors in lieu of a less-characterized media product, but this may not preclude their usage. Overall, our analyses of single cell heterogeneity show that our system is well positioned to further investigate the effects of both known and unknown physiological cues on PC differentiation and function.

One of the most important features we established with our CI-PCs was the ability to measure PC functional enrichment through simple soluble assays. We demonstrated sufficient functional enrichment in PCs such that enzymatic activity assays can detect stimulant-induced secretion of antimicrobials as well as the promotion of the ISC niche. Moreover, microbe co-culture assays with our enriched cells produce measurable and selective microbial growth modulation not observed using conventional organoids. Co-culture strains were chosen to demonstrate proof of concept of selective antimicrobial action and assess functionality compared to conventional organoids. Given the results showing selective modulation of bacterial growth, we believe that our system could serve as a tool to further probe host-microbe interaction in vitro. Furthermore, it would allow for investigations of both microbial mechanisms that elicit PC response (e.g., TLRs) and the properties of complex mixtures of secreted components, including multiple antimicrobial proteins.

## Conclusions

The generation of comprehensive cellular atlases from humans and model organisms will revolutionize our understanding of complex tissues [3]. Intestinal organoids have already proven their value in studying human and murine epithelial biology. However, to rigorously test hypotheses of basic biological or disease mechanism, it will be essential to have simple and reliable protocols for the generation of specialized subsets of cells which cannot be readily isolated from tissue. The representativeness of cell states present in organoids and the specialized cell types present in vivo [3] is an outstanding question with implications in mucosal immunology, developmental biology, and translational medicine. Our single-cell genomics approach provides compelling evidence that organoid-derived cell populations must be validated to ensure physiological relevance, and additionally provides a rational framework for identifying cell states and their potential upstream drivers to modulate cellular composition. This approach could enable advances beyond conventional organoid systems to provide an enriched highly specialized cell population that recapitulates important physiological functions of the intestinal epithelium and could represent an improvement in in vitro PC culture for the purposes of high-throughput screening, the study of host-microbe interactions, bioengineering (e.g., precision gene editing), and the identification of novel genetic candidates in PC function (e.g., *Nupr1*). With this framework, we illustrate the power and importance of rigorously characterizing the specialized cell types derived in organoids to those defined in ‘atlas-level’ surveys of the intestinal epithelium.

## Methods

### Mice for tissue isolation

Proximal small intestine was isolated from C57BL/6 mice of both sexes, aged between 3 and 6 months in all experiments.

### Bacteria strains

Cells were stored at –80 ºC and grown as follows. *E. coli* strain MG1655 was grown overnight in LB. For experiments, overnight cultures of MG1655 were resuspended in M9 supplemented with 0.4% glucose and 0.2% CASamino acids. *L. lactis* strain MG1363 was grown in M17 media supplemented with 0.5% glucose, and *E. faecalis* strain V583 was grown in Brain Heart Infusion media.

### Crypt culture, enrichment, and differentiation

Small intestinal crypts were cultured as previously described [59]. Briefly, crypts were resuspended in basal culture medium (Advanced DMEM/F12 with 2 mM GlutaMAX and 10 mM HEPES; Thermo Fisher Scientific) at a 1:1 ratio with Corning™ Matrigel™ Membrane Matrix – GFR (Fisher Scientific) and plated at the center of each well of 24-well plates. Following Matrigel polymerization, 500 μL of small intestinal crypt culture medium (basal media plus 100X N2 supplement, 50X B27 supplement; Life Technologies, 500X N-acetyl-L-cysteine; Sigma-Aldrich) supplemented with growth factors EGF (E; 50 ng/mL, Life Technologies), Noggin (N; 100 ng/mL, PeproTech), and R-spondin 1 (R; 500 ng/mL, PeproTech) and small molecules CHIR99021 (C; 3 μM, LC Laboratories) and valproic acid (V; 1 mM, Sigma-Aldrich) was added to each well. ROCK inhibitor Y-27632 (Y; 10 μM, R&D Systems) was added for the first 2 days of culture. Cells were cultured at 37 °C with 5% CO_2_, and cell culture medium was changed every other day. After 6 days of culture, crypt organoids were isolated from Matrigel by mechanical dissociation. Isolated organoids were resuspended in TrypLE Express (Life Tech) to dissociate into single cells, then replated in Matrigel with ENR + CV + Y media for 2 days. Cells were once again passaged, either into freezing media (Life Tech) for cryopreservation or replated at approximately 200 organoids per well (24-well plate) for ISC-enriched organoid expansion. ISC-enriched organoids were passaged or differentiated every 6 days in the ENR + CV condition. To differentiate, cells were passaged as previously described, and crypt culture medium containing growth factors ENR only or ENR + CD (DAPT, 10 μM; Sigma-Aldrich) was added to each well.

### RNA extraction and qRT-PCR

Organoids were isolated from Matrigel in 24-well plates following culture as previously described, and pellets were lysed in TRI reagent with RNA extracted according to the manufacturer’s protocol (T9424, Sigma). Resulting RNA pellets were dissolved in UltraPure water and cDNA synthesis was performed using QuantiTect Reverse Transcription Kit (Qiagen). qPCR reactions were performed using TaqMan Universal Master Mix II (no UNG), pre-designed TaqMan probes (Additional file 9: Table S5), and 50 ng of sample cDNA (LifeTech). Reactions were performed using an Applied Biosystems 7900HT system. qPCR results were analyzed using RQ manager 1.2 software to obtain CT values used for relative quantification to the housekeeping gene *Hprt*.

### Confocal imaging of whole cell clusters

ISC-enriched cell clusters (ENR + CV) suspended in 40 μL of Matrigel were seeded onto round coverslips inside a 24-well plate. Cells were treated with ENR + CD, ENR + CV, or ENR as previously described. At day 6, organoids were rinsed (PBS0 3X) and fixed on the coverslips by incubating with 4% paraformaldehyde (PFA) for 30 min at room temperature (RT). Gels were blocked and permeabilized by incubating at RT for 1 hour with 0.1% Triton X-100 and 5% Powerblock in PBS0. Organoids were stained for DEFA and LYZ by incubating with rat anti-mouse Crp1 (Ayabe Lab clone 77-R63, 5 μg/mL, 50X) and rabbit anti-human Lyz (Dako, RRID: AB_2341230, 200X) primary antibodies diluted to 10 μg/mL in staining solution (0.1% Triton X-100 and 10X Powerblock in PBS0) overnight at 4 °C, followed by secondary antibodies Alexa Fluor 647 anti-Rabbit IgG (RRID: AB_2563202, 400X) and Alexa Fluor 488 anti-Rat IgG (RRID: AB_2563120, 400X) diluted in staining solution for 1 h at RT. Actin was stained with Alexa Fluor 555 Phalloidin (40X) for 20 min, followed by staining of the nucleus with 3 μM DAPI for 5 min. Coverslips were mounted onto slides with Vectashield and imaged within 5 days using an Olympus FV2000 confocal microscope. Whole organoid confocal microscopy images were processed and analyzed using ImageJ. To determine the PC purity percentage, the ImageJ Point Picker plugin was used to count the number of nuclei to determine the total number of cells and to count the number of DEFA- and LYZ-containing PCs across all z-slices. To investigate cell polarity in whole organoids, individual cells were selected using ImageJ and mean area intensity within selected cell areas was computed in each z-slice throughout the depth of the image across every channel imaged.

### High-resolution single-cell imaging

Cell clusters were harvested and rinsed (basal culture media 3X) to remove Matrigel as previously described. Isolated clusters were resuspended in TrypLE Express and incubated at 37 °C for 20 min to dissociate into single cells, then rinsed (basal culture media 2X) and resuspended in PBS containing magnesium and calcium. Pre-coated poly-L-lysine coverslips (Fisher Scientific) were placed into wells of a 24-well plate, a cell suspension containing approximately 50,000 cells per well was added to each well, and the plate was centrifuged at 700 rcf for 5 min. PBS supernatant was removed from the wells, and the cells attached to the coverslips were fixed by incubating with 4% PFA for 30 min at RT. After each step, cells were rinsed (PBS 2–5 min 3X). Cells were blocked and permeabilized by incubating at RT for 30 min with permeabilization solution and stained with DEFA and LYZ by incubating with rat anti-mouse Crp1 (Ayabe Lab clone 77-R63, 5 μg/mL, 50X) and rabbit anti-human Lyz (Dako, RRID: AB_2341230, 200X) primary antibodies diluted in staining solution overnight at 4 °C. Secondary antibodies Alexa Fluor 647 anti-Rabbit IgG (RRID: AB_2563202, 400X) and Alexa Fluor 488 anti-Rat IgG (RRID: AB_2563120, 400X) diluted in staining solution were incubated with the coverslips for 1 h at RT. Actin was stained with Alexa Fluor 555 Phalloidin incubated for 20 min at RT, and the nucleus was stained with DAPI by incubating at RT for 5 mins. Coverslips were mounted on to slides with Vectashield and imaged within 48 h using an Applied Precision DeltaVision Microscope.

### Flow cytometry

Cell clusters were isolated from Matrigel as previously described and resuspended in TrypLE Express at 37 °C for 20 min to dissociate into single cells. Dissociated cells were centrifuged at 300 *g* for 3 min at 4 °C. The pellet was resuspended in FACS buffer (1% FBS in PBS, Thermo Fisher Scientific) and strained into a 5 mL filter cap tube using a 40 μm filter. The cell suspension was transferred to a flow prep microcentrifuge tube and centrifuged at 300 rcf for 3 min. Cell pellets were resuspended in a Zombie violet dye (BioLegend, 100X) in FACS buffer for viability staining followed by 1% PFA fixation for 20 min at RT. Pellets were permeabilized for 20 min at RT with staining buffer (0.5% Tween-20 in FACS buffer, Sigma), and co-stained with rabbit anti-human FITC-Lyz (Dako, RRID: AB_578661, 100X) and rat anti-mouse APC-CD24 (Biolegend, RRID: AB_2565650, 100X) antibodies diluted in staining buffer for 45 min at RT. Flow cytometry was performed using a BD LSR II HTS (BD; Koch Institute Flow Cytometry Core at MIT). Initial settings and laser voltages were determined with unstained, single channel stains or secondary-only controls (data not shown). Flow cytometry data was analyzed using FlowJo v10.7 software. Briefly, gating was performed as seen in Additional file 3: Figure S1F by removing doubles and debris, then selecting the BV421^–^ (viable) cell population; within this population, gating was based on LYZ^–^ and CD24^–^ populations.

### Lysozyme functional secretion assay

Lysozyme secretion was measured using a Lysozyme Assay Kit (EnzChek; Thermo Fisher). Briefly, cells suspended in Matrigel in 24-well plates were washed (basal culture media 3X) and either supplemented with 500 μL of basal culture media or basal culture media plus 10 μM CCh (Sigma Aldrich) for 24 h at 37 °C. Following stimulation, culture plates were spun at high speed (> 2000 *g*) for 5 min at RT to pellet cell debris and loose Matrigel, and 25 μL of conditioned supernatant was removed from the top of each well and quantified as per the manufacturer’s protocol.

### Quantification of cell viability, apoptosis, and cytotoxicity

To track proliferation and cell viability, DNA content was quantified over the course of differentiation and CCh stimulation using a CyQUANT Cell Proliferation Assay Kit (Thermo Fisher) as per the manufacturer’s protocol. Briefly, culture media was aspirated from each well, and the wells were washed (PBS 3X). Gels were then mechanically dissociated into PBS, the contents transferred into a Falcon tube, centrifuged at 300 rcf for 3 min at 4 °C, and the pellet resuspended in PBS to wash. Tubes were centrifuged at 300 rcf for 5 min at 4 °C, and the pellet was resuspended in 1 mL of assay working solution (20X cell-lysis buffer, 400X GR dye in DI water); 200 μL of samples and DNA standards were plated in triplicate in a black 96-well plate, shaken for 5 min, then fluorescence was measured on a plate reader (480 nm/520 nm).

For ENR/ENR + CD co-culture, ISC-enriched organoids (ENR + CV) were differentiated in ENR and ENR + CD and isolated as previously described. The cell pellets were counted and resuspended in basal culture medium, mixed at 0:100, 25:75, 50:50, 75:25, and 100:0% ENR:ENR + CD ratios (number of clusters), and plated as previously described in Matrigel in a 96-well plate at approximately 50 clusters per well in ENR media. After 24 and 48 h of co-culture, viability versus cytotoxicity and caspase activation were assessed using ApoTox-Glo Triplex Assay (Promega) according to the manufacturer’s protocol. Briefly, 20 μL of ‘V/C reagent’ (10 μL each of GF-AFC and bis-AAF-R110 substrates in 2.0 mL of assay buffer) were added to all wells and mixed by orbital shaking at 500 rpm for 30 s. After 30 min of incubation at 37 °C, fluorescence was measured on a plate reader (400 nm/505 nm for viability and 485 nm/520 nm for cytotoxicity). Caspase-Glo 3/7 reagent (100 μL) was then added to all wells and mixed by orbital shaking at 500 rpm for 30 s. After 30 min of incubation at RT, luminescence was measured on a plate reader.

### Bacteria co-culture

For bacteria co-culture, ISC-enriched cells (ENR + CV) were differentiated in ENR and ENR + CD as previously described. After 6 days of differentiation, cell clusters were isolated as previously described. The cell pellet was resuspended in basal culture medium and plated in suspension in a 96-well plate at approximately 150 clusters per well. A 1:1 volume of bacteria in respective media (see ‘Bacterial strains’, above; in exponential growth, as confirmed by plate reader OD) was added, and bacterial growth was measured by serial plating (CFU) after a 4 h incubation. Results for bacteria co-culture were normalized to no cell (bacteria only) controls.

### Mass spectrometry proteomics sample preparation, sequencing, and quantification

Organoid cell pellets were isolated from Matrigel with mechanical dissociation and washed (cold PBS 5X) to remove residual extracellular protein. Proteins were extracted from cell pellets with 8 M urea (Sigma), reduced with 5 mM DTT (Thermo Fisher Pierce) for 45 min, alkylated with 10 mM IAA (Sigma) for 45 min in the dark, and double digested with both Lysyl Endopeptidase ‘LysC’ (Wako) and trypsin (Promega) overnight at RT. A small aliquot of cellular lysate was removed from each sample for protein quantification via the Pierce™ BCA Protein Assay Kit (Pierce). After proteolytic digestion, the samples were quenched using formic acid to a final concentration of 1.0% and subsequently desalted on 10 mg OASIS HLB solid phase columns (Waters).

From each condition (*n* = 8), 50 μg aliquots of the Ng KD dried tryptic peptides were reconstituted in 100 mM HEPES at pH 8.0 to a final concentration of 1.0 mg/mL. The peptides were labeled with TMT-10 isobaric mass tag reagent according to the manufacturer’s instructions (ThermoFisher Scientific). The peptides were labeled at a 1:8 ratio of peptide to TMT reagent, followed by 1 h incubation at RT with bench-top shaking at 850 rpm. After incubation, a 1.0 μg aliquot of labeled tryptic peptide was removed from each labeled condition, desalted with C18 stage tips, and analyzed via LC-MS/MS using a Thermo Fisher Q Exactive Plus Hybrid Mass Spectrometer (QE-Plus) coupled to a Thermo Fisher EASY-nLC 1000 liquid chromatograph to ensure isobaric label incorporation ≥ 95%. An additional 1.0 μg of labeled tryptic peptide was removed from each channel, mixed together, desalted on a C18 stage tip, and analyzed via LC-MS to ensure equal relative protein loads. During these quality control steps, the labeled peptides were stored, unquenched, at –80 °C. After validation, each channel was quenched with a 5% hydroxylamine solution to a final sample concentration of 0.3% to quench any unbound isobaric tags. The corresponding eight channels were mixed together for a total amount of 400 μg of labeled tryptic peptides. The labeled peptide mixture was dried down in a speedvac and subsequently desalted on 30 mg of OASIS HLB solid phase column (Waters).

The dried, labeled peptides were fractionated into 24 fractions by basic reversed-phase using an Agilent Zorbax 300 Å, 4.6 mm × 250 mm Extend-C18 column on an Agilent 1100 Series HPLC instrument (Agilent Technologies) to decrease sample complexity and increase the dynamic range of detection. Solvent A (2% acetonitrile, 5 mM ammonium formate, pH 10), and a non-linear increasing concentration of solvent B (90% acetonitrile, 5 mM ammonium formate, pH 10) was used as the mobile phase with a flow rate of 1 mL/min through the column. A non-linear gradient with increasing percentages of solvent B with four different slopes was used (0% for 7 min; 0% to 16% in 6 min; 16% to 40% in 60 min; 40% to 44% in 4 min; 44% to 60% in 5 min; 60% for 14 min), and the eluted peptides were collected in a Whatman polypropylene 2 mL 96-well plate (Whatman). The 96 fractions were concatenated down to 25 fractions.

The global proteome (25 fractions) was analyzed by LC-MS/MS using the same system described above. Peptides were separated at a flow rate of 200 nL min^–1^ on a capillary column (Picofrit with a 10-μm tip opening and 75 μm diameter, New Objective, PF360-75-10-N-5) packed at the Broad Institute with 20 cm of C18 1.9 μm silica beads (1.9 μm ReproSil-Pur C18-AQ medium, Dr. Maisch GmbH, r119.aq). Injected peptides were separated at a flow rate of 200 nL min^–1^ with a linear 84 min gradient from 100% solvent A (3% acetonitrile, 0.1% formic acid) to 30% solvent B (90% acetonitrile, 0.1% formic acid), followed by a linear 9 min gradient from 30% solvent A to 90% solvent B for a total of 110 min. The QE-Plus instrument was operated in the data-dependent mode acquiring higher-energy collisional dissociation tandem mass spectrometry (HCD MS/MS) scans (Resolution = 35,000) for TMT-10 on the 12 most abundant ions using an MS1 ion target of 3 × 10^6^ ions and an MS2 target of 5 × 10^4^ ions. The maximum ion time used for the MS/MS scans was 120 ms; the HCD-normalized collision energy was set to 31; the dynamic exclusion time was set to 20 s, and the peptide-match preferred setting was enabled.

### Protein and peptide identification and quantification

Peptide spectrum matching and protein identification was performed using Agilent Technologies SM software package (developed at the Broad Institute). In SM, FDRs are calculated at three different levels, namely spectrum, distinct peptide, and distinct protein. Peptide FDRs are calculated in SM using essentially the same pseudo-reversal strategy evaluated by Elias and Gygi [60] and shown to perform the same as library concatenation. A false distinct protein ID occurs when all the distinct peptides that group together to constitute a distinct protein have a deltaForwardReverseScore ≤ 0. We adjusted the settings to provide peptide FDR of 1–2% and protein FDR of 0–1%*.* SM also carries out sophisticated protein grouping using the methods previously described [61]. Only proteins with more than two peptides and at least two TMT ratios in each replicate were counted as being identified and quantified. Additionally, we added the capability to flag potentially unreliable TMT quantification results based on detection of more than one precursor in the selection window for MS/MS. The precursor ion flagging was similar to that recently reported [62], but was carried out post data acquisition. As an output, SM generates protein and peptide reports for downstream differential regulation, pathway, and network analysis. Prior to comprehensive differential marker, pathway, and network analysis with the SM-generated protein reports, we ensured that the data was of high quality and has been properly normalized. The first level of normalization was accomplished by guaranteeing that an equivalent amount of peptide (50 μg per) was labeled for each of the 10 TMT channels. Once the SM reports were generated, we calculated the median ratios for each of the channels where the denominator of the ratio was a predetermined TMT channel signifying the control condition. The underlying assumption was that the null distribution is centered at zero in log2 space. Therefore, in this step of normalization, we normalized the median log2 ratio for each ratio column so that the median log2 ratio was zero. To robustly and confidently detect real differential peptides and proteins in the TMT-labeled experiment, we performed a moderated *t* test [63, 64]. Unlike the standard *t* test, which is not robust for small numbers of samples, the moderated *t* test uses an empirical Bayes approach that ‘moderates’ variance estimates for peptides (i.e., shrunk towards a common value), thereby significantly improving the stability of variance estimates for individual peptides. The *p* values reported by the moderated *t* test were adjusted for multiple testing using the Benjamini–Hochberg FDR method [64].

### Proteome pathway and network analysis

Using the identified and quantified proteins from the TMT-10 labeling experiment, multiple pathway and network analyses were performed. Sample correlations were represented as r-values and determined using GraphPad Prism version 7.0a. To elucidate potential transcriptional drivers of proteome structure, we performed GSEA v3.0b2 [37, 38] using the full rank-ordered proteome against the transcription factor target gene set database (v5.2 MSigDB) [39], then performed enrichment map visualization using GSEA-P-based implementation and Cytoscape v3.4.0 [40, 41], with a moderately conservative cutoff (*p* < 0.005 and FDR < 0.075) and an overlap coefficient of 0.2. To assess the functional and compartmental functions associated with the ENR + CD-enriched proteome and ENR-enriched proteome, we used DAVID v6.8 [65, 66] and the gene ontology database, looking only at experimentally verified associations within biological processes, cellular compartments, and molecular function against a background set of all 8015 quantified proteins.

### Single-cell RNA-sequencing

A single-cell suspension was obtained from organoids cultured under ENR + CV, ENR, and ENR + CD conditions for 6 days as described above. We utilized the Seq-Well platform for massively parallel scRNA-seq to capture transcriptomes of single cells on barcoded mRNA capture beads. Full methods on implementation of this platform are available in Gierahn et al. [28]. In brief, 20,000 cells from one organoid condition were loaded onto one array containing 100,000 barcoded mRNA capture beads. The loaded arrays containing cells and beads were then sealed using a polycarbonate membrane with a pore size of 0.01 μm, which allows for exchange of buffers but retains biological molecules confined within each microwell. Subsequent exchange of buffers allows for cell lysis, transcript hybridization, and bead recovery before performing reverse transcription en masse. Following reverse transcription and exonuclease treatment to remove excess primers, PCR amplification was carried out using KAPA HiFi PCR Mastermix with 2000 beads per 50 μL reaction volume. Six libraries (totaling 12,000 beads) were then pooled and purified using Agencourt AMPure XP beads (Beckman Coulter, A63881) by a 0.6X SPRI followed by a 0.7X SPRI and quantified using Qubit hsDNA Assay (Thermo Fisher). Libraries were constructed using the Nextera Tagmentation method on a total of 800 pg of pooled cDNA library from 12,000 recovered beads. Tagmented and amplified sequences were purified at a 0.6X SPRI ratio yielding library sizes with an average distribution of 650–750 base pairs in length as determined using the Agilent hsD1000 Screen Tape System (Agilent Genomics). Arrays were sequenced with an Illumina 75 Cycle NextSeq500/550v2 kit at a final concentration of 2.8 pM. The read structure was paired end with Read 1 starting from a custom read 1 primer containing 20 bases with a 12 bp cell barcode and 8 bp UMI and Read 2 being 50 bases containing transcript information (Additional file 10: Table S6 for primers used).

### Single-cell RNA-sequencing computational pipelines and analysis

Read alignment was performed as in Macosko et al. [67]. Briefly, for each NextSeq sequencing run, raw sequencing data was converted to demultiplexed FASTQ files using bcl2fastq2 based on Nextera N700 indices corresponding to individual samples/arrays. Reads were then aligned to mm10 genome using the Galaxy portal maintained by the Broad Institute for Drop-Seq alignment using standard settings. Individual reads were tagged according to the 12 bp barcode sequencing and the 8 bp UMI contained in Read 1 of each fragment. Following alignment, reads were binned onto 12 bp cell barcodes and collapsed by their 8 bp UMI. Digital gene expression matrices (e.g., cell by gene tables) for each sample were obtained from quality filtered and mapped reads and UMI-collapsed data, were deposited in GSE100274, and were utilized as input into Seurat (https://github.com/satijalab/seurat) for further analysis [68].

To analyze ENR + CV, ENR, and ENR + CD organoids together, we merged UMI matrices across all genes detected in any condition and generated a matrix retaining all cells with at least 1000 UMI detected. This table was then utilized to setup the Seurat object in which any cell with at least 400 unique genes was retained and any gene expressed in at least five cells was retained. The object was initiated with log-normalization, scaling, and centering set to True. Before performing dimensionality reduction, data was subset to include cells with less than 8000 UMI, and a list of 1676 most variable genes was generated by including genes with an average normalized and scaled expression value greater than 0.14 and with a dispersion (variance/mean) greater than 0.4. The total number of ENR + CV, ENR, and ENR + CD cells included in the analysis was 985, 2544, and 2382, respectively, with quality metrics for nGene, nUMI, and percentage of ribosomal and mitochondrial genes reported in Additional file 8: Figure S4. We then performed principal component analysis over the list of variable genes. For both clustering and t-stochastic neighbor embedding (tSNE), we utilized the first 12 principal components based on the elbow method, as upon visual inspection of genes contained within, each contributing to important biological processes of intestinal cells. We used FindClusters with a resolution of 1.35 and 1000 iterations of tSNE to identify 14 clusters across the three input samples. To identify genes which defined each cluster, we performed a ROC test implemented in Seurat with a threshold set to an AUC of 0.60.

### Transcriptional scoring

To determine the fractional contribution to a cell’s transcriptome of a gene list, we summed the total log(scaled UMI + 1) expression values for genes within a list of interest and divided by the total amount of scaled UMI detected in that cell giving a proportion of a cell’s transcriptome dedicated to producing those genes. From our proteomic screen, we took a list of upregulated proteins (249) or downregulated proteins (212) that were detected within our single-cell RNA-sequencing data. To determine the relationship to in vivo PCs and EECs, we took reference data from two Seq-Well experiments run on epithelial cells dissociated from the ileal region of the small intestine of two C57BL/6 J mice run in separate experiments. The ileum was first rinsed in 30 mL of ice cold PBS and allowed to settle. The segment was then sliced with scissors and transferred to 10 mL of epithelial cell solution (HBSS Ca/Mg-Free 10 mM EDTA, 100 U/mL penicillin, 100 μg/mL streptomycin, 10 mM HEPES, 2% FCS (ThermoFisher)) freshly supplemented with 200 μL of 0.5 M EDTA. The epithelial separation from the underlying lamina propria was performed for 15 min at 37 °C in a rotisserie rack with end-over-end rotation. The tube was then removed and placed on ice immediately for 10 min before shaking vigorously 15 times. Visual macroscopic inspection of the tube at this point yielded visible epithelial sheets, and microscopic examination confirmed the presence of single-layer sheets and crypt-villus structures. The epithelial fraction was spun down at 400 *g* for 7 min and resuspended in 1 mL of epithelial cell solution before transferring to a 1.5 mL Eppendorf tube to minimize the time spent centrifuging. Cells were spun down at 800 *g* for 2 min and resuspended in TrypLE Express for 5 min in a 37 °C bath followed by gentle trituration with a P1000 pipette. Cells were spun down at 800 *g* for 2 min and resuspended in ACK lysis buffer (ThermoFisher) for 3 min on ice to remove red blood cells and dying cells. Cells were spun down at 800 *g* for 2 min and resuspended in 1 mL of epithelial cell solution and placed on ice for 3 min before triturating with a P1000 pipette and filtering into a new Eppendorf through a 40 μm cell strainer (Falcon/VWR). Cells were spun down at 800 *g* for 2 min and then resupended in 200 μL of epithelial cell solution and placed on ice for counting. Single-cell RNA-seq data was then generated as described in the ‘Single-cell RNA-sequencing’ and ‘Single-cell RNA-sequencing computational pipelines and analysis’ sections in Methods. To generate PC and EEC signatures, we ran unbiased SNN-graph based clustering, performed a ROC test, identified the two mature PC and EEC clusters, and reported all genes with an AUC above 0.60, using all genes with an AUC above 0.65 for scoring within each cluster (gene lists in Additional file 1: Table S1), representing any gene with enrichment in PCs and EECs. These lists capture genes which are enriched in PC (*Lyz*-high) and EECs (*Chga*-high) and separate them from the rest of the cells present in intestinal epithelium. For pathway analysis, we inspected curated gene lists deposited in the GSEA platform and used KEGG-derived Wnt and Reactome-derived Notch and Respiratory Electron Transport Chain signatures (Additional file 2: Table S2). In vivo transcription factors for PCs and EECs were determined by matching the PC and EEC signature gene sets with transcription factors from the Riken Transcription Factor Database (TFdb - http://genome.gsc.riken.jp/TFdb/), and then including only those TFs which were robustly identified in the proteome dataset.

### Quantification and statistical analysis

All statistical analyses were performed using GraphPad Prism v7.0a, Seurat implemented in RStudio, and Agilent Technologies Spectrum Mill software package. All graphs with *n* > 6 show mean ± SEM, unless otherwise noted, whereas graphs with *n* < 6 show mean and individual replicate values. Unpaired two-tail *t* test and two-way ANOVA with Dunnett’s multiple comparison test (reported as adj. *p* value) were used to assess statistical significance as appropriate and, unless otherwise noted, * indicates *p* < 0.05, ** *p* < 0.01 *** *p* < 0.001, **** *p* < 0.0001, and ns non-significant. In each experiment, tissues were isolated from multiple mice housed in the same facility with each mouse providing tissue designated as a distinct biological donor: *n* = 3 donor-averaged values of four technical replicates for data reported in Fig. 2b; *n* = 3 donor of two technical replicates for data reported in Fig. 2e (with exception of day 8, *n* = 2 donor-averaged values of two technical replicates) and Additional file 3: Figure S1F; *n* = 4 (two technical replicates from two biological donors each) for data reported in Figs. 2f–h, and Additional file 4: Figure S2; *n* = 1 biological donor for in vitro data reported in Figs. 3 and 4 and Additional file 7: Figure S3 and Additional file 8: Figure S4; *n* = 8 single-well replicates from one and five biological donors for data reported in Fig. 5a, b and c, respectively; *n* = 13 co-culture well replicates randomly selected without replacement from four donors for data reported in Fig. 5d; *n* = 6 well replicates (two per three biological donors) in Fig. 5e; *n* = 3 biological donors in Fig. 6c, and *n* = 5 biological donors in Fig. 6d. For scores in single-cell data, we report effect sizes in addition to statistical significance as an additional metric for the magnitude of the effect observed. The calculation was performed as Cohen’s *d*, where effect size *d* = (Mean_1_-Mean_2_)/(SD pooled).

## Additional files

Additional file 1: **Table S1.** Derived gene list of the top defining genes from in vivo ileal small intestine PCs and EECs captured on the Seq-Well platform. (XLSX 355 kb)

Additional file 2: **Table S2.** Reference gene lists used in single-cell analyses. (XLSX 24 kb)

Additional file 3: **Figure S1.** Image analysis of cell clusters and flow cytometry. A Bright-field microscopy after 6 days of ENR + CD culture shows annular morphology and darkened lumen of cell clusters consistent with presence of granule-rich cells. B Percentage of total cells that are LYZ+ and DEFA+ following 6 days of ENR, ENR + CV, and ENR + CD culture (from cell counting of whole clusters) (*n* = 3 minimum biological replicates, one-way ANOVA with multiple comparison test versus ENR, **** adj. *p* < 0.0001). C Collapsed z-stack of whole cluster with individual cells highlighted (1–3) following 6 days of ENR + CD, stained for LYZ and DEFA and counterstained with DAPI and for actin (phalloidin). (1–3) Normalized mean-area intensity versus z-axis depth profiles of representative individual LYZ+/DEFA+ co-staining cells. D Representative flow cytometry of ENR and ENR + CD at 6 days with distinct populations of CD24+ and LYZ+ cells indicative of phenotypic PCs. E Representative gating for flow cytometry, including removal of doublets and non-viable cells in final gating. F Percentage of viable cells (membrane impermeable) over time of ENR versus ENR + CD culture. (PDF 3217 kb)

Additional file 4: **Figure S2.** Proteomic pipeline, sample-to-sample comparison, and insights from the in vitro PC proteome. A Schematic of proteomic analysis for samples: culture, collection, lysis, reduction and alkylation, proteolytic digestion, labeling of peptides with isobaric mass tag reagents (Tandem Mass Tags, TMT10-plex; Thermo), off-line fractionation by basic reverse phase chromatography, analysis of fractions by LC-MS/MS, identification of peptides and proteins using Spectrum Mill software (Agilent), and statistical analysis of the resulting data (moderated *t* test) to identify confidently differential proteins. B Proteome sample correlation between all biological (*n* = 2) and technical (*n* = 2/biological) replicates. C ENR + CD-enriched proteins are well-annotated in the gene ontology (GO) database and show robust enrichment for functions and compartments of secretory cells determined by fold enrichment vs. FDR using DAVID. D ENR-enriched proteins are well annotated in the GO database and show enrichment for functions and compartments of transcriptionally and translationally active cells determined by fold enrichment vs. FDR using DAVID. e GSEA enrichment map of transcription factors linked to ENR + CD- and ENR-enriched proteins following a moderately conservative cutoff of *p* < 0.005, FDR < 0.075, and overlap coefficient of 0.2. (PDF 483 kb)

Additional file 5: **Table S3.** Detected and quantified in vitro Proteome. (XLSX 919 kb)

Additional file 6: **Table S4.** Complete list of DAVID enrichments. (XLSX 51 kb)

Additional file 7: **Figure S3.** Quality metrics for single-cell RNA sequencing. A Total gene number of cells maintained in analyses with a lower cutoff of *n* = 400 unique genes per cell. Total unique molecular identifiers (UMIs) used as the basis for cell-by-gene tables collapsed to UMI as input into Seurat with the lower bound representing *n* = 400 unique genes and the upper bound representing 8000 UMIs. Note: Clusters ENR + CV-3, ENR + CV-4, and ENR-1 had significantly higher levels of genes and UMIs and, intriguingly, were also the three clusters with the highest levels of Lgr5 (see Fig. 5a), indicating that stem cells may contain larger contents of RNA, as they are in a biosynthetic state before differentiation and maturation. B Violin plot of expression contribution to a cell’s transcriptome of mitochondrial and ribosomal genes across identified subsets. (PDF 1153 kb)

Additional file 8: **Figure S4**. Signaling pathways and processes associated with in vitro PC enrichment (Additional file 9: Table S5 for reference gene lists). A Violin plot of expression contribution to a cell’s transcriptome of Wnt pathway genes (Additional file 2: Table S2; activated by CHIR99021) across clusters as percent of transcriptome. B Violin plot of expression contribution to a cell’s transcriptome of Notch pathway genes (Additional file 2: Table S2; inhibited by DAPT) across clusters as percent of transcriptome. C Violin plot of expression contribution to a cell’s transcriptome of respiratory electron transport gene set (Additional file 2: Table S2) across clusters as percent of transcriptome. (PDF 888 kb)

Additional file 9 : **Table S5.** TaqMan gene expression assays used for qRT-PCR. (DOCX 13 kb)

Additional file 10: **Table S6.** SeqWell reverse transcription and library preparation primers. (DOCX 13 kb)

## References

[1] Clevers H (2016). Modeling development and disease with organoids. Cell.

[2] Prakadan SM, Shalek AK, Weitz DA (2017). Scaling by shrinking: empowering single-cell “omics” with microfluidic devices. Nat Rev Genet.

[3] Haber AL, Biton M, Rogel N, Herbst RH, Shekhar K, Smillie C (2017). A single-cell survey of the small intestinal epithelium. Nature.

[4] Grün D, Lyubimova A, Kester L, Wiebrands K, Basak O, Sasaki N (2015). Single-cell messenger RNA sequencing reveals rare intestinal cell types. Nature..

[5] The HCA Consortium. The human cell atlas white paper. 2017; https://www.humancellatlas.org/files/HCA_WhitePaper_18Oct2017.pdf. Accessed 29 May 2018.

[6] Tanay A, Regev A (2017). Scaling single-cell genomics from phenomenology to mechanism. Nature.

[7] Satija R, Shalek AK. Heterogeneity in immune responses: From populations to single cells. Trends Immunol. 2014;35:219–29. 10.1016/j.it.2014.03.004 10.1016/j.it.2014.03.004 PMC4035247 24746883

[8] Lancaster MA, Knoblich JA. Organogenesis in a dish: Modeling development and disease using organoid technologies. Science. 2014;345:1247125. 10.1126/science.1247125 25035496

[9] Schwank G, Koo BK, Sasselli V, Dekkers JF, Heo I, Demircan T (2013). Functional repair of CFTR by CRISPR/Cas9 in intestinal stem cell organoids of cystic fibrosis patients. Cell Stem Cell..

[10] Drost J, van Boxtel R, Blokzijl F, Mizutani T, Sasaki N, Sasselli V (2017). Use of CRISPR-modified human stem cell organoids to study the origin of mutational signatures in cancer. Science.

[11] Molodecky NA, Soon IS, Rabi DM, Ghali WA, Ferris M, Chernoff G (2012). Increasing incidence and prevalence of the inflammatory bowel diseases with time, based on systematic review. Gastroenterology.

[12] Wehkamp J, Salzman NH, Porter E, Nuding S, Weichenthal M, Petras RE (2005). Reduced Paneth cell α-defensins in ileal Crohn’s disease. Proc Natl Acad Sci U S A.

[13] Ireland H, Houghton C, Howard L, Winton DJ (2005). Cellular inheritance of a Cre-activated reporter gene to determine Paneth cell longevity in the murine small intestine. Dev Dyn.

[14] Sato T, van Es JH, Snippert HJ, Stange DE, Vries RG, van den Born M (2011). Paneth cells constitute the niche for Lgr5 stem cells in intestinal crypts. Nature.

[15] Clevers HC, Bevins CL (2013). Paneth cells: maestros of the small intestinal crypts. Annu Rev Physiol.

[16] Xavier RJ, Podolsky DK (2007). Unravelling the pathogenesis of inflammatory bowel disease. Nature.

[17] Khor B, Gardet A, Xavier RJ (2011). Genetics and pathogenesis of inflammatory bowel disease. Nature.

[18] Liu T-C, Gurram B, Baldridge MT, Head R, Lam V, Luo C (2016). Paneth cell defects in Crohn’s disease patients promote dysbiosis. JCI Insight.

[19] Adolph TE, Tomczak MF, Niederreiter L, Ko H-J, Böck J, Martinez-Naves E (2013). Paneth cells as a site of origin for intestinal inflammation. Nature.

[20] Kobayashi KS, Chamaillard M, Ogura Y, Henegariu O, Inohara N, Nuñez G (2005). Nod2-dependent regulation of innate and adaptive immunity in the intestinal tract. Science.

[21] Kaser A, Blumberg RS (2014). ATG16L1 Crohn’s disease risk stresses the endoplasmic reticulum of Paneth cells. Gut.

[22] Kaser A, Lee A-H, Franke A, Glickman JN, Zeissig S, Tilg H (2008). XBP1 links ER stress to intestinal inflammation and confers genetic risk for human inflammatory bowel disease. Cell.

[23] Farin HF, Karthaus WR, Kujala P, Rakhshandehroo M, Schwank G, Vries RGJ (2014). Paneth cell extrusion and release of antimicrobial products is directly controlled by immune cell-derived IFN-γ. J Exp Med.

[24] Wilson SS, Tocchi A, Holly MK, Parks WC, Smith JG (2014). A small intestinal organoid model of non-invasive enteric pathogen-epithelial cell interactions. Mucosal Immunol.

[25] Foulke-Abel J, In J, Yin J, Zachos NC, Kovbasnjuk O, Estes MK (2016). Human enteroids as a model of upper small intestinal ion transport physiology and pathophysiology. Gastroenterology.

[26] Moon C, VanDussen KL, Miyoshi H, Stappenbeck TS (2013). Development of a primary mouse intestinal epithelial cell monolayer culture system to evaluate factors that modulate IgA transcytosis. Mucosal Immunol.

[27] Basak O, Beumer J, Wiebrands K, Seno H, van Oudenaarden A, Clevers H (2017). Induced quiescence of Lgr5+ stem cells in intestinal organoids enables differentiation of hormone-producing enteroendocrine cells. Cell Stem Cell..

[28] Gierahn TM, Wadsworth MH, Hughes TK, Bryson BD, Butler A, Satija R (2017). Seq-Well: portable, low-cost RNA sequencing of single cells at high throughput. Nat Methods.

[29] Yin X, Farin HF, van Es JH, Clevers H, Langer R, Karp JM (2014). Niche-independent high-purity cultures of Lgr5+ intestinal stem cells and their progeny. Nat Methods.

[30] Yin X, Mead BE, Safaee H, Langer R, Karp JM, Levy O (2016). Engineering stem cell organoids. Cell Stem Cell.

[31] McLean WJ, Yin X, Lu L, Lenz DR, McLean D, Langer R (2017). Clonal expansion of Lgr5-positive cells from mammalian cochlea and high-purity generation of sensory hair cells. Cell Rep.

[32] van Es JH, Jay P, Gregorieff A, van Gijn ME, Jonkheer S, Hatzis P (2005). Wnt signalling induces maturation of Paneth cells in intestinal crypts. Nat Cell Biol.

[33] VanDussen KL, Carulli AJ, Keeley TM, Patel SR, Puthoff BJ, Magness ST (2012). Notch signaling modulates proliferation and differentiation of intestinal crypt base columnar stem cells. Development.

[34] Tian H, Biehs B, Chiu C, Siebel CW, Wu Y, Costa M (2015). Opposing activities of notch and wnt signaling regulate intestinal stem cells and gut homeostasis. Cell Rep.

[35] Buczacki SJ, Zecchini HI, Nicholson AM, Russell R, Vermeulen L, Kemp R (2013). Intestinal label-retaining cells are secretory precursors expressing Lgr5. Nature.

[36] von Furstenberg RJ, Gulati AS, Baxi A, Doherty JM, Stappenbeck TS, Gracz AD (2011). Sorting mouse jejunal epithelial cells with CD24 yields a population with characteristics of intestinal stem cells. AJP Gastrointest Liver Physiol.

[37] Subramanian A, Tamayo P, Mootha VK, Mukherjee S, Ebert BL, Gillette MA (2005). Gene set enrichment analysis: A knowledge-based approach for interpreting genome-wide expression profiles. Proc Natl Acad Sci.

[38] Mootha VK, Lindgren CM, Eriksson K-F, Subramanian A, Sihag S, Lehar J (2003). PGC-1α-responsive genes involved in oxidative phosphorylation are coordinately downregulated in human diabetes. Nat Genet.

[39] Xie X, Lu J, Kulbokas EJ, Golub TR, Mootha V, Lindblad-Toh K (2005). Systematic discovery of regulatory motifs in human promoters and 3’ UTRs by comparison of several mammals. Nature.

[40] Merico D, Isserlin R, Stueker O, Emili A, Bader GD. Enrichment map: a network-based method for gene-set enrichment visualization and interpretation. PLoS One [Internet]. 2010;5:e13984. Available from: http://www.ncbi.nlm.nih.gov/pubmed/21085593 10.1371/journal.pone.0013984 PMC2981572 21085593

[41] Shannon P, Markiel A, Ozier O, Baliga NS, Wang JT, Ramage D, et al. Cytoscape: a software environment for integrated models of biomolecular interaction networks. Genome Res. 2003;13:2498–504. 10.1101/gr.1239303 PMC403769 14597658

[42] Stringari C, Edwards RA, Pate KT, Waterman ML, Donovan PJ, Gratton E (2012). Metabolic trajectory of cellular differentiation in small intestine by Phasor Fluorescence Lifetime Microscopy of NADH. Sci Rep.

[43] Rodríguez-Colman MJ, Schewe M, Meerlo M, Stigter E, Gerrits J, Pras-Raves M (2017). Interplay between metabolic identities in the intestinal crypt supports stem cell function. Nature.

[44] Ayabe T, Satchell DP, Wilson CL, Parks WC, Selsted ME, Ouellette AJ (2000). Secretion of microbicidal alpha-defensins by intestinal Paneth cells in response to bacteria. Nat Immunol.

[45] Kanamori M, Konno H, Osato N, Kawai J, Hayashizaki Y, Suzuki H (2004). A genome-wide and nonredundant mouse transcription factor database. Biochem Biophys Res Commun.

[46] Jia SN, Lin C, Chen DF, Li AQ, Dai L, Zhang L (2016). The transcription factor p8 regulates Autophagy in response to palmitic acid stress via a mammalian target of rapamycin (mTOR)-independent signaling pathway. J Biol Chem.

[47] Grasso D, Bintz J, Lomberk G, Molejon MI, Loncle C, Garcia MN (2015). Pivotal role of the chromatin protein Nupr1 in Kras-induced senescence and transformation. Sci Rep.

[48] Cano CE, Hamidi T, Sandi MJ, Iovanna JL (2011). Nupr1: The Swiss-knife of cancer. J Cell Physiol.

[49] Imielinski M, Baldassano RN, Griffiths A, Russell RK, Annese V, Dubinsky M (2009). Common variants at five new loci associated with early-onset inflammatory bowel disease. Nat Genet.

[50] Santofimia-Castaño P, Rizzuti B, Pey ÁL, Soubeyran P, Vidal M, Urrutia R, et al. Intrinsically disordered chromatin protein NUPR1 binds to the C-terminal region of Polycomb RING1B. Proc Natl Acad Sci. 2017; 10.1073/pnas.1619932114. 10.1073/pnas.1619932114 PMC5547589 28720707

[51] Neira JL, Bintz J, Arruebo M, Rizzuti B, Bonacci T, Vega S (2017). Identification of a drug targeting an intrinsically disordered protein involved in pancreatic adenocarcinoma. Sci Rep..

[52] Gulati AS, Shanahan MT, Arthur JC, Grossniklaus E, von Furstenberg RJ, Kreuk L (2012). Mouse background strain profoundly influences Paneth cell function and intestinal microbial composition. PLoS One.

[53] Bevins CL, Salzman NH (2011). Paneth cells, antimicrobial peptides and maintenance of intestinal homeostasis. Nat Rev Microbiol.

[54] Zhang Q, Pan Y, Yan R, Zeng B, Wang H, Zhang X (2015). Commensal bacteria direct selective cargo sorting to promote symbiosis. Nat Immunol.

[55] Cunliffe RN, Rose FR, Keyte J, Abberley L, Chan WC, Mahida YR (2001). Human defensin 5 is stored in precursor form in normal Paneth cells and is expressed by some villous epithelial cells and by metaplastic Paneth cells in the colon in inflammatory bowel disease. Gut.

[56] Beumer J, Clevers H (2016). Regulation and plasticity of intestinal stem cells during homeostasis and regeneration. Development.

[57] Janda CY, Dang LT, You C, Chang J, de Lau W, Zhong ZA (2017). Surrogate Wnt agonists that phenocopy canonical Wnt and β-catenin signalling. Nature.

[58] Gjorevski N, Sachs N, Manfrin A, Giger S, Bragina ME,Ordóñez-Morán P et al. Designer matrices for intestinal stem cell and organoid culture. Nature 2016;539:560–564. 10.1038/nature20168. 10.1038/nature20168 27851739

[59] Sato T, Vries RG, Snippert HJ, van de Wetering M, Barker N, Stange DE (2009). Single Lgr5 stem cells build crypt-villus structures in vitro without a mesenchymal niche. Nature.

[60] Elias JE, Gygi SP. In: Hubbard SJ, Jones AR, editors. Target-Decoy Search Strategy for Mass Spectrometry-Based Proteomics. Totoewa: Humana Press; 2010. p. 55–71. http://link.springer.com/10.1007/978-1-60761-444-9. 10.1007/978-1-60761-444-9_5 PMC2922680 20013364

[61] Nesvizhskii AI, Aebersold R (2005). Interpretation of Shotgun proteomic data. Mol Cell Proteomics.

[62] Phanstiel DH, Brumbaugh J, Wenger CD, Tian S, Probasco MD, Bailey DJ (2011). Proteomic and phosphoproteomic comparison of human ES and iPS cells. Nat Methods.

[63] Smyth GK (2004). Linear Models and Empirical Bayes Methods for Assessing Differential Expression in Microarray Experiments. Stat Appl Genet Mol Biol.

[64] Benajmini Y, Hochberg Y (1995). Controlling the false discovery rate: a practical and powerful approach to multiple testing. J R Stat Soc B.

[65] Huang DW, Sherman BT, Lempicki RA (2009). Systematic and integrative analysis of large gene lists using DAVID bioinformatics resources. Nat Protoc.

[66] Huang DW, Sherman BT, Lempicki RA (2009). Bioinformatics enrichment tools: Paths toward the comprehensive functional analysis of large gene lists. Nucleic Acids Res.

[67] Macosko EZ, Basu A, Satija R, Nemesh J, Shekhar K, Goldman M (2015). Highly parallel genome-wide expression profiling of individual cells using nanoliter droplets. Cell.

[68] Satija R, Farrell JA, Gennert D, Schier AF, Regev A (2015). Spatial reconstruction of single-cell gene expression data. Nat Biotechnol.

