# Supplementary material for: Harnessing single-cell genomics to improve the physiological fidelity of organoid-derived cell types
Source: BMC Biol. 2018 Jun 5;16:62. doi: 10.1186/s12915-018-0527-2 (PMC5989470; doi:10.1186/s12915-018-0527-2)
Supplement: Supplementary file 4 — Figure S2. Proteomic pipeline, sample-to-sample comparison, and insights from the in vitro PC proteome. A Schematic of proteomic analysis for samples: culture, collection, lysis, reduction and alkylation, proteolytic digestion, labeling of peptides with isobaric mass tag reagents (Tandem Mass Tags, TMT10-plex; Thermo), off-line fractionation by basic reverse phase chromatography, analysis of fractions by LC-MS/MS, identification of peptides and proteins using Spectrum Mill software (Agilent), and statistical analysis of the resulting data (moderated t test) to identify confidently differential proteins. B Proteome sample correlation between all biological (n = 2) and technical (n = 2/biological) replicates. C ENR + CD-enriched proteins are well-annotated in the gene ontology (GO) database and show robust enrichment for functions and compartments of secretory cells determined by fold enrichment vs. FDR using DAVID. D ENR-enriched proteins are well annotated in the GO database and show enrichment for functions and compartments of transcriptionally and translationally active cells determined by fold enrichment vs. FDR using DAVID. e GSEA enrichment map of transcription factors linked to ENR + CD- and ENR-enriched proteins following a moderately conservative cutoff of p < 0.005, FDR < 0.075, and overlap coefficient of 0.2. (PDF 483 kb) [file 12915_2018_527_MOESM4_ESM.pdf]

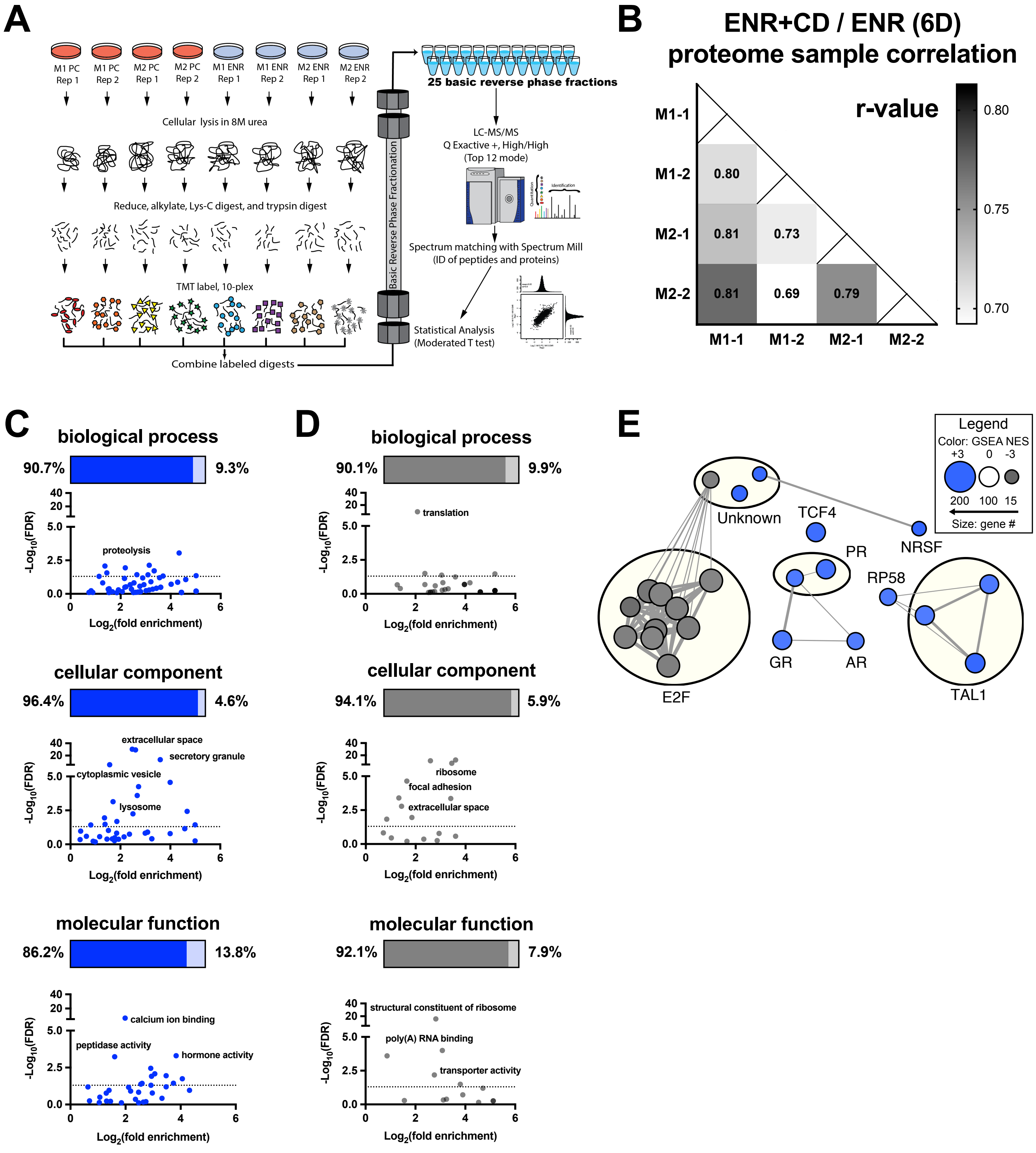
A
M1 PC Rep 1
M1 PC Rep 2
M2 PC Rep 1
M2 PC Rep 2
M1 ENR Rep 1
M1 ENR Rep 2
M2 ENR Rep 1
M2 ENR Rep 2
Cellular lysis in 8M urea
Reduce, alkylate, Lys-C digest, and trypsin digest
TMT label, 10-plex
Combine labeled digests
Basic Reverse Phase Fractionation
25 basic reverse phase fractions
LC-MS/MS
Q Exactive +, High/High
(Top 12 mode)
Spectrum matching with Spectrum Mill
(ID of peptides and proteins)
Statistical Analysis
(Moderated T test)
B
ENR+CD / ENR (6D)
proteome sample correlation
r-value
M1-1
M1-2
M2-1
M2-2
0.80
0.81
0.73
0.81
0.69
0.79
0.80
0.75
0.70
C
biological process
90.7%
9.3%
proteolysis
-Log10(FDR)
Log2(fold enrichment)
cellular component
96.4%
4.6%
extracellular space
secretory granule
cytoplasmic vesicle
lysosome
molecular function
86.2%
13.8%
calcium ion binding
peptidase activity
hormone activity
D
biological process
90.1%
9.9%
translation
cellular component
94.1%
5.9%
ribosome
focal adhesion
extracellular space
molecular function
92.1%
7.9%
structural constituent of ribosome
poly(A) RNA binding
transporter activity
E
Legend
Color: GSEA NES
+3
0
-3
200
100
15
Size: gene #
Unknown
TCF4
NRSF
PR
RP58
E2F
GR
AR
TAL1
