# Supplementary figures and images for: Harnessing single-cell genomics to improve the physiological fidelity of organoid-derived cell types
Source: BMC Biol. 2018 Jun 5;16:62. doi: 10.1186/s12915-018-0527-2 (PMC5989470; doi:10.1186/s12915-018-0527-2)

**A**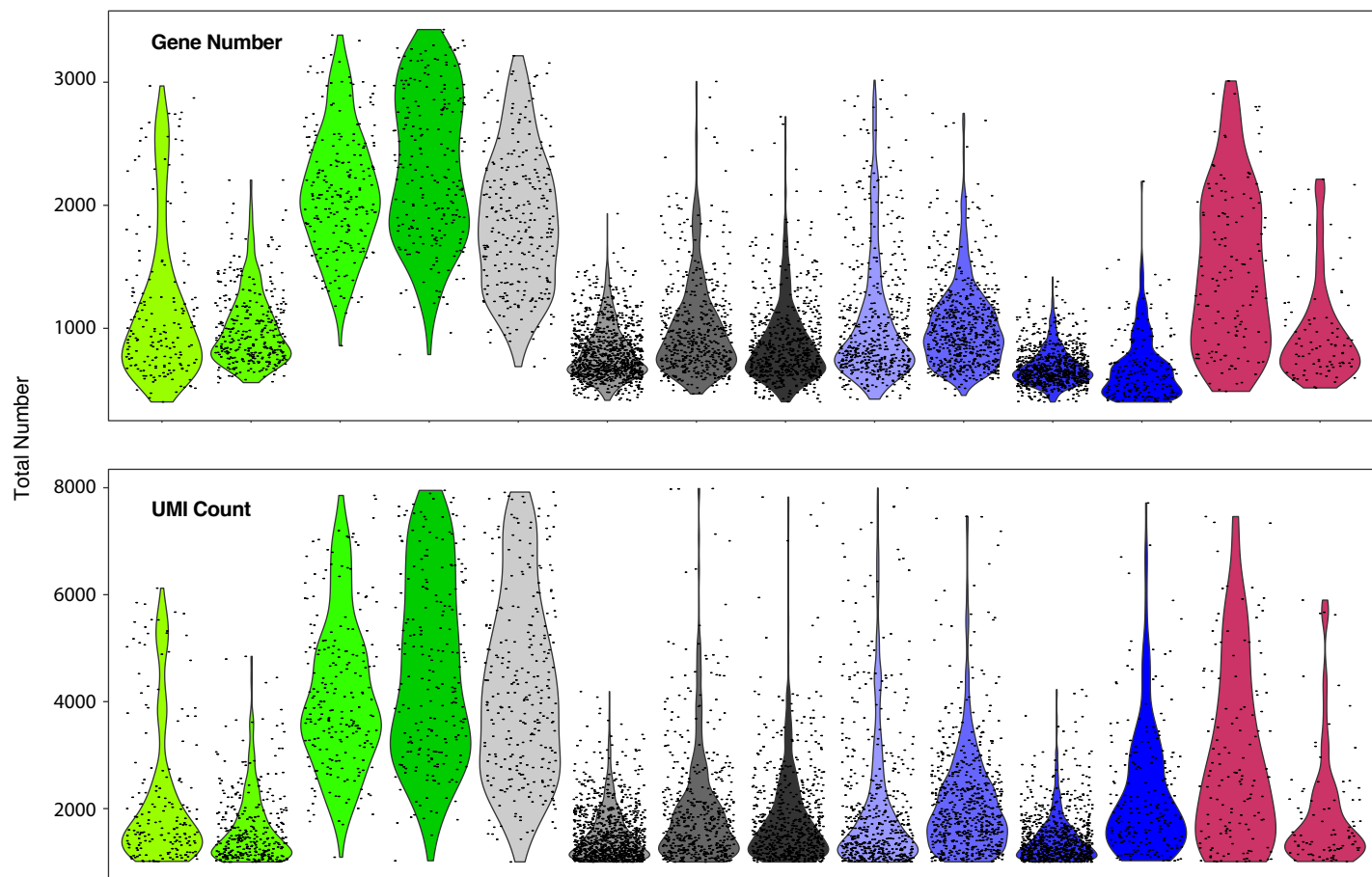**B**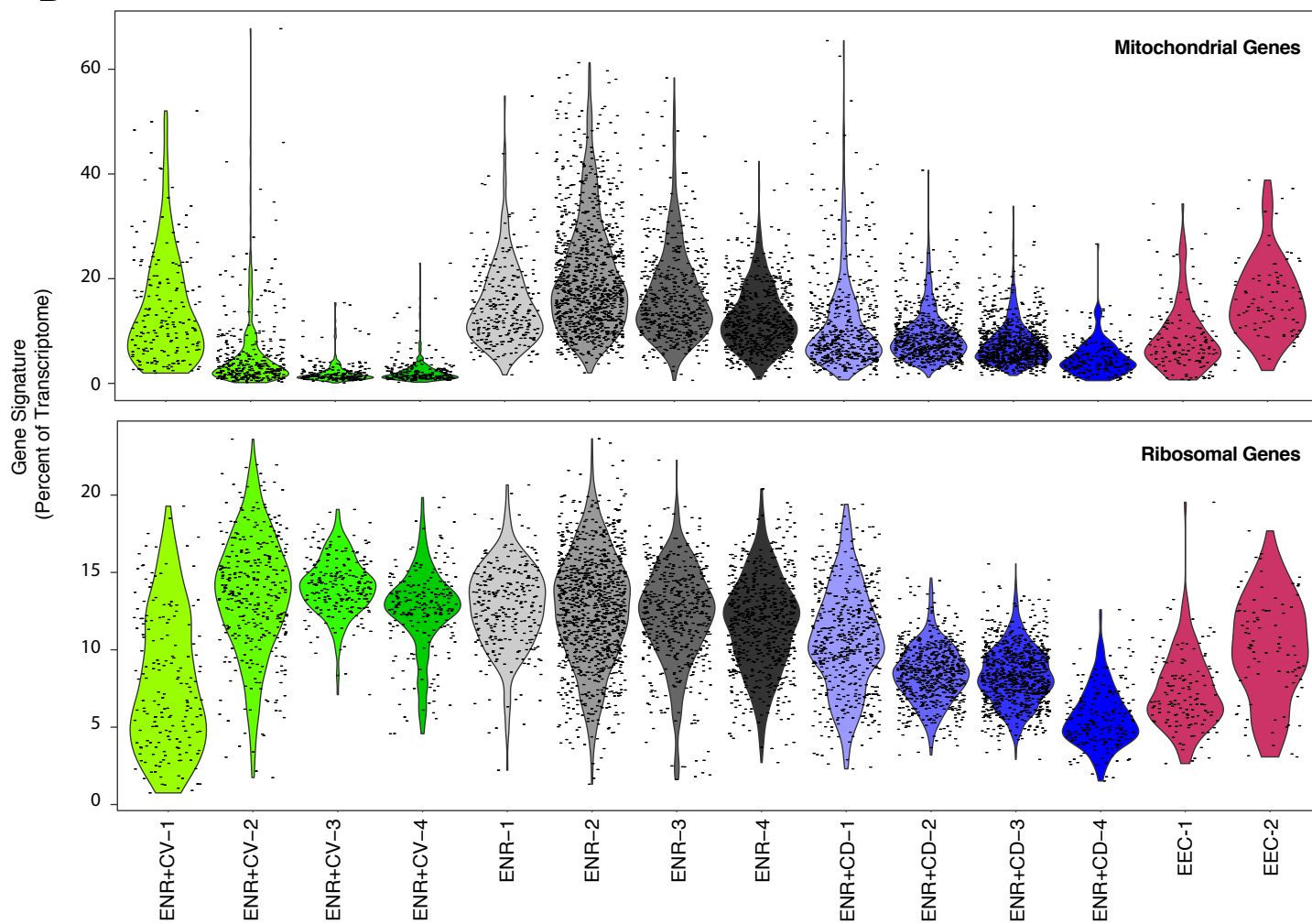

Supplement: Supplementary file 7 — Figure S3. Quality metrics for single-cell RNA sequencing. A Total gene number of cells maintained in analyses with a lower cutoff of n = 400 unique genes per cell. Total unique molecular identifiers (UMIs) used as the basis for cell-by-gene tables collapsed to UMI as input into Seurat with the lower bound representing n = 400 unique genes and the upper bound representing 8000 UMIs. Note: Clusters ENR + CV-3, ENR + CV-4, and ENR-1 had significantly higher levels of genes and UMIs and, intriguingly, were also the three clusters with the highest levels of Lgr5 (see Fig. 5a), indicating that stem cells may contain larger contents of RNA, as they are in a biosynthetic state before differentiation and maturation. B Violin plot of expression contribution to a cell’s transcriptome of mitochondrial and ribosomal genes across identified subsets. (PDF 1153 kb) [file 12915_2018_527_MOESM7_ESM.pdf]

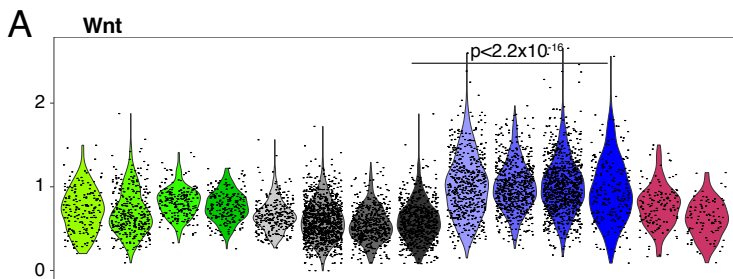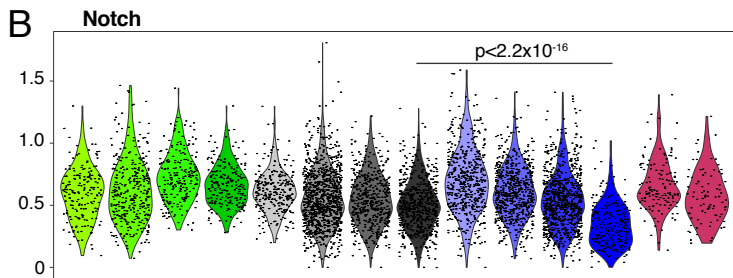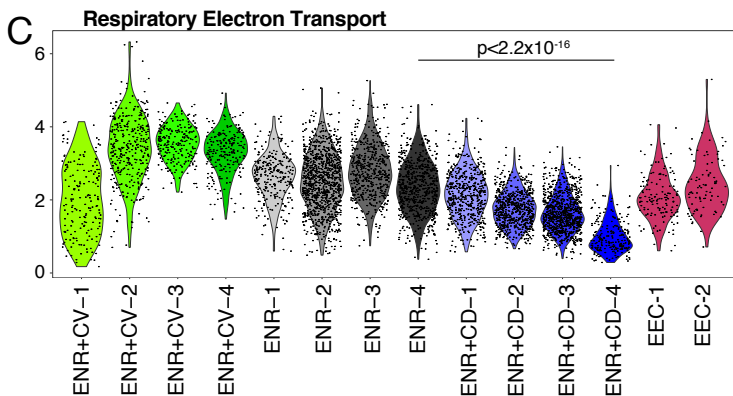

Supplement: Supplementary file 8 — Figure S4. Signaling pathways and processes associated with in vitro PC enrichment (Additional file 9: Table S5 for reference gene lists). A Violin plot of expression contribution to a cell’s transcriptome of Wnt pathway genes (Additional file 2: Table S2; activated by CHIR99021) across clusters as percent of transcriptome. B Violin plot of expression contribution to a cell’s transcriptome of Notch pathway genes (Additional file 2: Table S2; inhibited by DAPT) across clusters as percent of transcriptome. C Violin plot of expression contribution to a cell’s transcriptome of respiratory electron transport gene set (Additional file 2: Table S2) across clusters as percent of transcriptome. (PDF 888 kb) [file 12915_2018_527_MOESM8_ESM.pdf]
