# Supplementary material for: Harnessing single-cell genomics to improve the physiological fidelity of organoid-derived cell types
Source: BMC Biol. 2018 Jun 5;16:62. doi: 10.1186/s12915-018-0527-2 (PMC5989470; doi:10.1186/s12915-018-0527-2)
Supplement: Supplementary file 9 — : Table S5. TaqMan gene expression assays used for qRT-PCR. (DOCX 13 kb) [file 12915_2018_527_MOESM9_ESM.docx]

**Table S5: TaqMan gene expression assays used for qRT-PCR**

| **Organoid Cell Markers** |
| --- |
| *Lgr5*: Mm00438890_m1 |
| *Defa1*: Mm02524428_g1 |
| *Lyz1*: Mm00657323_m1 |
| *Mmp7*: Mm00487724_m1 |
|  |
| **Housekeeping Gene** |
| *Hprt*: Mm01545399_m1 |
