# Supplementary material for: Harnessing single-cell genomics to improve the physiological fidelity of organoid-derived cell types
Source: BMC Biol. 2018 Jun 5;16:62. doi: 10.1186/s12915-018-0527-2 (PMC5989470; doi:10.1186/s12915-018-0527-2)
Supplement: Supplementary file 10 — Table S6. SeqWell reverse transcription and library preparation primers. (DOCX 13 kb) [file 12915_2018_527_MOESM10_ESM.docx]

**Table S6: SeqWell reverse transcription and library preparation primers**

| **Oligo Name** | **Sequence (5’ to 3’)** |
| --- | --- |
| Barcoded Bead Sequence | 5’–Bead–Linker-TTTTTTTAAGCAGTGGTATCAAC  GCAGAGTACJJJJJJJJJJJJNNNNNNNN  TTTTTTTTTTTTTTTTTTTTTTTTTTTTTT-3’ |
| Template Switching Oligo (TSO) | AAGCAGTGGTATCAACGCAGAGTGAATrGrGrG |
| SMART PCR Primer (ISPCR) | AAGCAGTGGTATCAACGCAGAGT |
| New P5-SMART PCR Hybrid Oligo | AATGATACGGCGACCACCGAGATCTACACGCCT  GTCCGCGGAAGCAGTGGTATCAACGCAGAGT* A*C |
| Nextera N700 (i7 generic, insert BC oligo sequence in [i7] position) | CAAGCAGAAGACGGCATACGAGAT[i7]GTCTCGTGGGCTCGG |
| Nextera N700_BC5 (ENR) | AAGTAGAG |
| Nextera N700_BC7 (ENR+CD) | ACACGATC |
| Custom Read 1 Primer | GCCTGTCCGCGGAAGCAGTGGTATCAACGCAG AGTAC |
