# Supplementary material for: Harnessing single-cell genomics to improve the physiological fidelity of organoid-derived cell types
Source: BMC Biol. 2018 Jun 5;16:62. doi: 10.1186/s12915-018-0527-2 (PMC5989470; doi:10.1186/s12915-018-0527-2)
Supplement: Supplementary file 3 — Figure S1. Image analysis of cell clusters and flow cytometry. A Bright-field microscopy after 6 days of ENR + CD culture shows annular morphology and darkened lumen of cell clusters consistent with presence of granule-rich cells. B Percentage of total cells that are LYZ+ and DEFA+ following 6 days of ENR, ENR + CV, and ENR + CD culture (from cell counting of whole clusters) (n = 3 minimum biological replicates, one-way ANOVA with multiple comparison test versus ENR, **** adj. p < 0.0001). C Collapsed z-stack of whole cluster with individual cells highlighted (1–3) following 6 days of ENR + CD, stained for LYZ and DEFA and counterstained with DAPI and for actin (phalloidin). (1–3) Normalized mean-area intensity versus z-axis depth profiles of representative individual LYZ+/DEFA+ co-staining cells. D Representative flow cytometry of ENR and ENR + CD at 6 days with distinct populations of CD24+ and LYZ+ cells indicative of phenotypic PCs. E Representative gating for flow cytometry, including removal of doublets and non-viable cells in final gating. F Percentage of viable cells (membrane impermeable) over time of ENR versus ENR + CD culture. (PDF 3217 kb) [file 12915_2018_527_MOESM3_ESM.pdf]

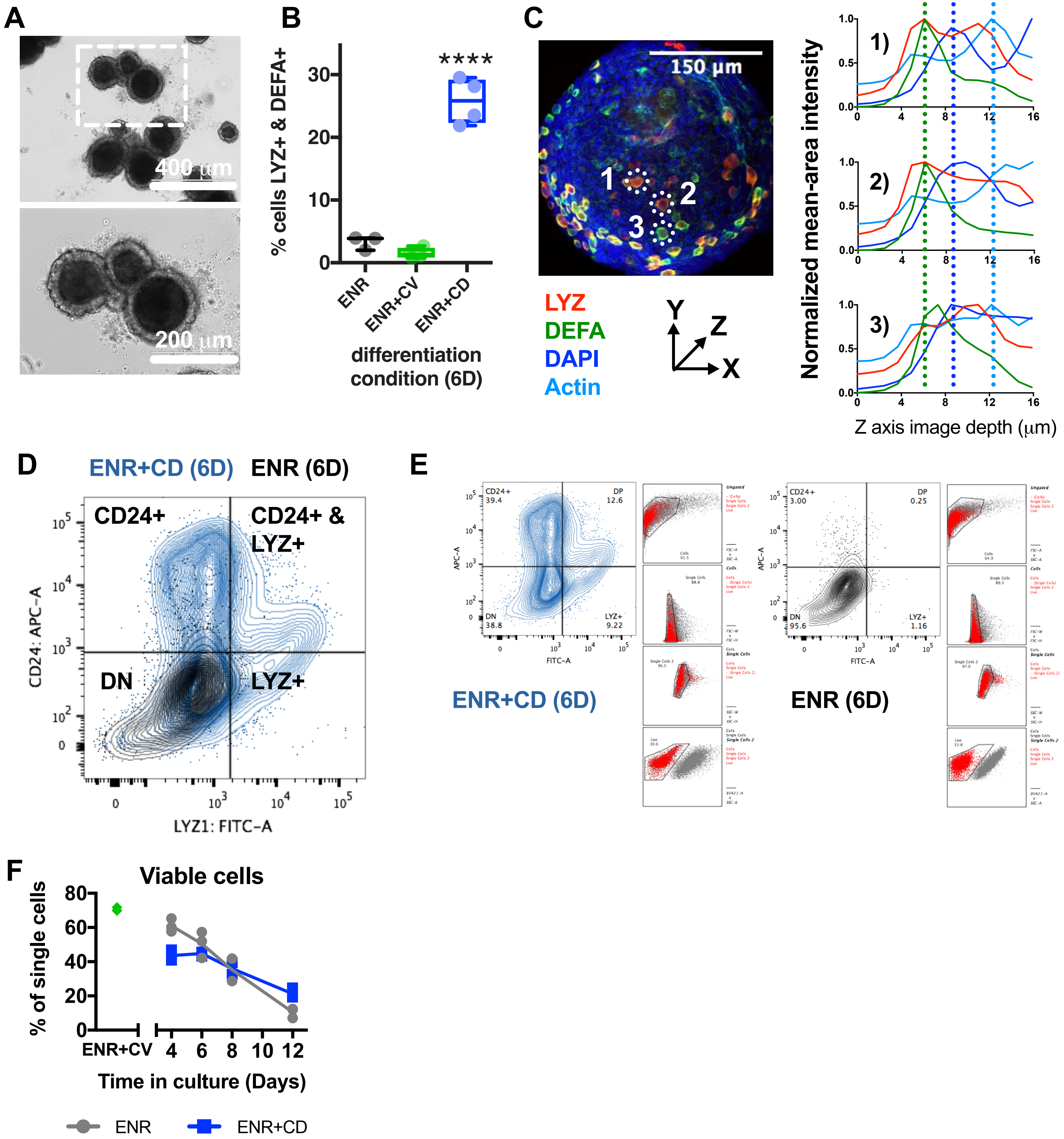
A
400 µm
200 µm
B
% cells LYZ+ & DEFA+
****
ENR
ENR+CV
ENR+CD
differentiation condition (6D)
C
150 µm
1
2
3
LYZ
DEFA
DAPI
Actin
Y
Z
X
Normalized mean-area intensity
1)
2)
3)
Z axis image depth (µm)
D
ENR+CD (6D)
ENR (6D)
CD24+
CD24+ & LYZ+
DN
LYZ+
CD24: APC-A
LYZ1: FITC-A
E
CD24+ 39.4
DP 12.6
DN 38.8
LYZ+ 9.22
APC-A
FITC-A
CD24+ 3.00
DP 0.25
DN 95.6
LYZ+ 1.16
ENR+CD (6D)
ENR (6D)
F
Viable cells
% of single cells
ENR+CV
Time in culture (Days)
ENR
ENR+CD
